# Supplementary material for: Vocal biomarker predicts fatigue in people with COVID-19: results from the prospective Predi-COVID cohort study
Source: BMJ Open. 2022 Nov 22;12(11):e062463. doi: 10.1136/bmjopen-2022-062463 (PMC9684280; doi:10.1136/bmjopen-2022-062463)
Supplement: Supplementary data [file bmjopen-2022-062463supp002.pdf]

Supplementary Online Material 2. VGG19 extracted features from participants' audio recordings

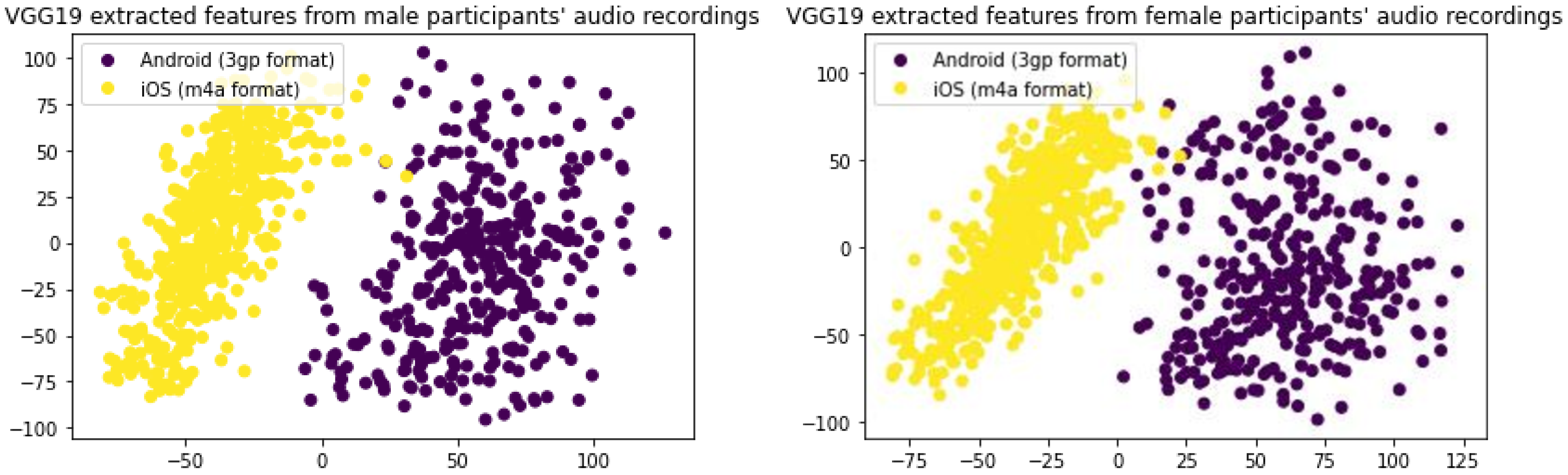

The scatter plot of the 250 relevant components given by PCA reduction revealed two distinct clusters. These two groups appeared to characterize the audio formats, m4a (iOS users) and 3gp (Android users).

It was consequently hypothesized that our data was heterogeneous and that it would be preferable to fit the models with each audio format independently.
