# Supplementary material for: Vocal biomarker predicts fatigue in people with COVID-19: results from the prospective Predi-COVID cohort study
Source: BMJ Open. 2022 Nov 22;12(11):e062463. doi: 10.1136/bmjopen-2022-062463 (PMC9684280; doi:10.1136/bmjopen-2022-062463)
Supplement: Author's manuscript [file bmjopen-2022-062463.draft_revisions.pdf]

BMJ Open is committed to open peer review. As part of this commitment we make the peer review history of every article we publish publicly available.

When an article is published we post the peer reviewers' comments and the authors' responses online. We also post the versions of the paper that were used during peer review. These are the versions that the peer review comments apply to.

The versions of the paper that follow are the versions that were submitted during the peer review process. They are not the versions of record or the final published versions. They should not be cited or distributed as the published version of this manuscript.

BMJ Open is an open access journal and the full, final, typeset and author-corrected version of record of the manuscript is available on our site with no access controls, subscription charges or pay-per-view fees (http://bmjopen.bmj.com).

If you have any questions on BMJ Open's open peer review process please email info.bmjopen@bmj.com

BMJ Open

Development of a vocal biomarker for fatigue monitoring in people with COVID-19

Journal:	*BMJ Open*
Manuscript ID	bmjopen-2022-062463
Article Type:	Original research
Date Submitted by the Author:	01-Mar-2022

Complete List of Authors:	Elbéji, Abir; Luxembourg Institute of Health, Department of Precision Health<br>Zhang, Lu; Luxembourg Institute of Health, Department of Precision Health<br>Higa, Eduardo; Luxembourg Institute of Health, Department of Precision Health<br>Fischer, Aurélie; Luxembourg Institute of Health, Departement of Population Health<br>Despotovic, Vladimir; Luxembourg Institute of Health, Department of Precision Health<br>Nazarov, Petr V.; Luxembourg Institute of Health, Department of Precision Health<br>Aguayo, Gloria; Luxembourg Institute of Health, Department of Population Health<br>Fagherazzi, Guy; Luxembourg Institute of Health, Department of Precision Health

Keywords:	COVID-19, Health informatics < BIOTECHNOLOGY & BIOINFORMATICS, Public health < INFECTIOUS DISEASES

SCHOLARONE™
Manuscripts

I, the Submitting Author has the right to grant and does grant on behalf of all authors of the Work (as defined in the below author licence), an exclusive licence and/or a non-exclusive licence for contributions from authors who are: i) UK Crown employees; ii) where BMJ has agreed a CC-BY licence shall apply, and/or iii) in accordance with the terms applicable for US Federal Government officers or employees acting as part of their official duties; on a worldwide, perpetual, irrevocable, royalty-free basis to BMJ Publishing Group Ltd ("BMJ") its licensees and where the relevant Journal is co-owned by BMJ to the co-owners of the Journal, to publish the Work in this journal and any other BMJ products and to exploit all rights, as set out in our licence.

The Submitting Author accepts and understands that any supply made under these terms is made by BMJ to the Submitting Author unless you are acting as an employee on behalf of your employer or a postgraduate student of an affiliated institution which is paying any applicable article publishing charge ("APC") for Open Access articles. Where the Submitting Author wishes to make the Work available on an Open Access basis (and intends to pay the relevant APC), the terms of reuse of such Open Access shall be governed by a Creative Commons licence – details of these licences and which Creative Commons licence will apply to this Work are set out in our licence referred to above.

Other than as permitted in any relevant BMJ Author's Self Archiving Policies, I confirm this Work has not been accepted for publication elsewhere, is not being considered for publication elsewhere and does not duplicate material already published. I confirm all authors consent to publication of this Work and authorise the granting of this licence.

For peer review only

Development of a vocal biomarker for fatigue monitoring in people with COVID-19

Abir Elbéji[1], Lu Zhang[2], Eduardo Higa[1], Aurélie Fischer[1], Vladimir Despotovic[2], Petr V. Nazarov[2], Gloria A. Aguayo[1], Guy Fagherazzi[1]

1. Deep Digital Phenotyping Research Unit. Department of Precision Health. Luxembourg Institute of Health, 1 A-B rue Thomas Edison, L-1445 Strassen, Luxembourg

2. Bioinformatics Platform. Luxembourg Institute of Health, 1A-B, rue Thomas Edison, L-1445 Strassen, Luxembourg

Reprints and correspondence:

Dr Guy Fagherazzi, PhD, ADR

Director of Department of Precision Health

Head of the Deep Digital Phenotyping Research Unit

Luxembourg Institute of Health

1 AB rue Thomas Edison, L-1445 STRASSEN, LUXEMBOURG

Phone: +352 6 21 58 95 54

Email: guy.fagherazzi@lih.lu

Website: https://ddp.lih.lu/

For peer review only - http://bmjopen.bmj.com/site/about/guidelines.xhtml

Abstract

Objective

To develop a vocal biomarker for fatigue monitoring in people with COVID-19.

Design Prospective cohort study.

Setting Predi-COVID data between May 2020 and May 2021.

Participants

A total of 1772 voice recordings was used to train an AI-based algorithm to predict fatigue, stratified by gender and smartphone's operating system (Android/iOS). The recordings were collected from 296 participants tracked for two weeks following SARS-CoV-2 infection.

primary and secondary outcome measures

Four machine learning algorithms (Logistic regression, k-nearest neighbors, support vector machine, and soft voting classifier) were used to train and derive the fatigue vocal biomarker. A t-test was used to evaluate the distribution of the vocal biomarker between the two classes (Fatigue and No fatigue).

Results

The final study population included 56% of women and had a mean (±SD) age of 40 (±13) years. Women were more likely to report fatigue (*P<.001*). We developed four models for Android female, Android male, iOS female, and iOS male users with a weighted AUC of 79%, 85%, 86%, 82%, and a mean Brier Score of 0.15, 0.12, 0.17, 0.12, respectively. The vocal biomarker derived from the prediction models successfully discriminated COVID-19 participants with and without fatigue (t-test *P<.001*).

Conclusions

This study demonstrates the feasibility of identifying and remotely monitoring fatigue thanks to voice. Vocal biomarkers, digitally integrated into telemedicine technologies, are expected to improve the monitoring of people with COVID-19 or Long-COVID.

ClinicalTrials.gov Identifier: NCT0438098

For peer review only - http://bmjopen.bmj.com/site/about/guidelines.xhtml

Strengths and limitations

-This is the first study supporting the hypothesis that fatigue can be accurately monitored based on voice in people with COVID-19.

-The analyses were based on a multi-lingual database of standardized voice recordings collected in real-life from people with confirmed SARS-CoV-2 infection as determined by PCR.

-There is no similar dataset available yet in the literature to replicate our findings.

-The vocal biomarker is trained on a binary outcome (Fatigue, Yes/No) and does not reflect the entire spectrum of fatigue severity. Further work should be performed in that direction.

For peer review only

Introduction

Coronavirus disease 2019 (COVID-19) is a global outbreak. More than 199 million confirmed cases of COVID-19 have been detected worldwide as of 4 August 2021, with more than 4 million deaths reported by the World Health Organization[1]. The worldwide population and healthcare systems have been greatly impacted by the COVID-19 pandemic. The pandemic has essentially put whole healthcare systems under pressure, requiring national or regional lockdowns[2]. Finding solutions that allow healthcare providers to focus on the more important and urgent patients, was, and still is, critical.

This outbreak continues to impact people, with many patients suffering from a range of acute symptoms, such as fatigue. Fatigue is a common symptom in patients with COVID-19 that can impact their quality of life, treatment adherence, and can be associated with numerous complications[3]. Recent findings showed that fatigue is a major symptom of the frequently reported Long-COVID syndrome. After recovering from the acute disease caused by the SARS outbreak, up to 60% of patients reported chronic fatigue 12 months later[4]. This supports the need for long-term monitoring solutions for these patients.

In general, fatigue can be of two types: physical and mental[5] experiencing lack of energy, inability to start and perform everyday activities, and lack of desire to do things. In the context of COVID-19, determinants of fatigue were categorized as both central and psychological factors, the latest might also be indirectly caused by pandemic-related fear and anxiety[6,7].

Fatigue affects men and women differently and has previously been shown to be reported differently in the two genders. Men and women have different anatomy and physiology, resulting in significant sex differences in fatigability[8].

Telemedicine, artificial intelligence (AI), and big data predictive analytics are examples of digital health technologies that have the potential to minimize the damaging effects of COVID-19 by improving responses to public health problems at a population level[9]. Using telemonitoring technologies to enable self-surveillance and remote monitoring of symptoms might therefore help to improve and personalize COVID-19 care delivery[10].

For peer review only - http://bmjopen.bmj.com/site/about/guidelines.xhtml

Voice is a promising source of digital data since it is rich, user-friendly, inexpensive to collect, and non-invasive, and can be used to develop vocal biomarkers that characterize disease states. Previous research was mostly conducted in the field of neurodegenerative diseases, such as Parkinson's disease[11] and Alzheimer's disease[12]. There are also studies that confirm the relation of voice disorders to fatigue, e.g., in Chronic Fatigue Syndrome (CFS). Neuromuscular, neuropsychological and hormonal dysfunction associated with CFS can influence the phonation and articulation, and alter tension, viscosity and thickness of the tissue of the larynx, tongue and lips, leading to decreased voice quality[13]. Increased fatigue affects voice characteristics, such as pitch, word duration[14] and timing of articulated sounds[15]. Vocal changes related to fatigue are more observed in consonant sounds that require a high average airflow[16].

In the context of the COVID-19 pandemic, respiratory sounds (e.g coughs, breathing, and voice) are also used as sources of information to develop COVID-19 screening tools[17,18,19]. However, no previous work has been devoted to investigating the association of voice with COVID-19 symptoms.

We hypothesized that there is an association between fatigue and voice in patients with COVID-19 and that it is possible to train an AI-based model to identify fatigue and subsequently generate a digital vocal biomarker for fatigue monitoring. We used data from the large hybrid prospective Predi-COVID cohort study to investigate this hypothesis.

Methods

Study design

This project uses data from the Predi-COVID study[20]. Predi-COVID is a hybrid cohort study that started in May 2020 in Luxembourg and involved participants who should meet all of the following requirements: (1) a signed informed consent form; (2) participants with confirmed SARS-CoV-2 infection as determined by PCR at one of Luxembourg's certified laboratories; and (3) 18 years and older.

This study combines data from the national surveillance system, which is used for virtually all COVID-19 positive patients. Biological sampling, electronic patient-

For peer review only

reported outcomes, and smartphone voice recording were collected to identify vocal biomarkers of respiratory syndromes and fatigue in this study. More details about the Predi-COVID study can be found elsewhere[20].

Health Inspection collaborators made the initial phone contact with potential participants. Those who consented to participate were contacted by a qualified nurse from the Clinical and Epidemiological Investigation Center (CIEC - Luxembourg Institute of Health), who outlined the study and arranged home or hospital visits.

Patient and Public Involvement

The Predi-COVID initiative was an emergency response from national research institutions grouped under 'Research Luxembourg' to fight the COVID-19 pandemic in Luxembourg and contribute to the general effort in the crisis. Therefore, for timing and safety reasons, patients with COVID-19 were not directly included to participate in the study design. However, the first participants included in Predi-COVID provided feedback on general workflow, data collection, questionnaires, and sampling, which was taken into account in an amendment to the protocol[20].

Data collection

Participants were followed for up to a year using a smartphone app to collect voice data. To ensure a minimum quality level, participants were asked to record it in a quiet environment while maintaining a certain distance from the microphone, and an audio example of what was required was also provided.

All the participants of this study were invited to record two audio types. The first, Type 1 audio, required participants to read paragraph 1 of article 25 of the Declaration of Human Rights[21], in their preferred language: French, German, English, or Portuguese; and the second, Type 2 audio, required them to hold the [a] vowel phonation without breathing for as long as they could (see Supplementary Online Material 1 for more details).

Predi-COVID collects data in conformity with the German Society of Epidemiology's best practices guidelines[22]. To draft the manuscript, we followed the TRIPOD criteria

For peer review only

for reporting AI-based model development and validation, as well as the corresponding checklist.

All Predi-COVID participants recruited between May 2020 and May 2021 who reported their fatigue status ("I feel well" as "No Fatigue" and "I am fatigued"/"I don't feel well" as "Fatigue") on the same day as the audio recordings during the 14 days of follow-up were included in this study[23]. As a result, several audio recordings for a single participant were available for both audio types[24].

Audio characteristics and vocal biomarker training

The audio recordings were collected in two formats, 3gp format (Android devices) and m4a format (iOS devices). Based on the smartphone's operating system and the user's gender (male/female), we trained one model for each category. This stratification was performed to minimize data heterogeneity and deal with sex as a potential confounding bias.

Audio pre-processing

All of the raw audio recordings were pre-processed (Figure 1). They were initially converted to .wav files, with audios lasting less than 2 seconds being excluded. Then, an audio clustering (DBSCAN) on basic features was performed (duration, the average, sum, and standard deviation of signal power, and fundamental frequency) to detect the outliers and exclude poor quality audios. Finally, peak normalization was used to boost the volume of quiet audio segments, and leading and trailing silences longer than 350 seconds were trimmed.

Feature extraction

We used transfer learning for the feature extraction process since it is adapted for small training databases[25]. Transfer learning is a technique where a model is constructed and trained with a set containing a large amount of data and then transfer and apply this learning to our dataset on top of it. It has the advantage of reducing the amount of data required while shortening training time and improving performance when compared to models built from scratch[26].

For peer review only - http://bmjopen.bmj.com/site/about/guidelines.xhtml

Convolutional neural networks require a fixed input size, whereas audio instances in our dataset were of variable length. To deal with this issue, Zero-padding was used to set the duration of each audio file to 50 seconds (the maximum length in our database). To raise the amount of information fed to the classifiers, type 1 and type 2 audios were concatenated and used as a single input to the learning models.

All the audio recordings were first resampled to 8kHz and then converted to Mel-spectrograms using the Librosa library in Python. The hop-length was 2048 samples, and the number of Mel coefficients was set to 196. The Mel spectrograms were passed through VGG19 convolutional neural network architecture provided by Keras, which was pre-trained on the ImageNet database[27]. This approach, presented in Figure 2, may be considered as a feature extraction step, as it converts audio recordings to 512 feature maps, each of a size 6x6, leading to a total of 18432 features.

This large number of features is computationally expensive. Principal Component Analysis (PCA)[28] is therefore used for dimensionality reduction and to select the number of relevant components explaining the maximum of the variance in the data.

For peer review only

Statistical analysis

We divided our data into “Fatigue” and “No Fatigue” groups based on the participant’s reported answers for the inclusion and daily fatigue assessment of Predi-COVID. To characterize participants, descriptive statistics were used, which included means, standard deviations for quantitative variables, and counts and percentages for qualitative variables. The two population groups (3gp (Android users) and m4a (iOS users)) were compared using a student test for continuous variables, and a χ2 test for categorical variables.

A 10-fold cross-validation procedure was conducted on the training cohort participants to evaluate four classification models (logistic regression (LR), k-nearest neighbors (KNN), support vector machine (SVM), and soft voting classifier (VC), scikit-learn implementation in Python) at different regularization levels via a grid search, with the following evaluation metrics: area under the ROC curve (AUC), accuracy, F1-score, precision, and recall. The Brier score was also used to evaluate the calibration of the selected models.

The predicted probability of being classified as fatigued from the best model was considered as our final vocal biomarker, which may be used as a quantitative metric to monitor fatigue.

We evaluated the vocal biomarker’s distribution in both classes (Fatigue and No Fatigue) and performed a t-test between the two groups.

Results

Study population characteristics

The final study population is composed of 296 participants of whom 165 were women (56%), with an average age of 40 years (SD = 13). To record both audio types,109 (37%) participants utilized Android smartphones (3gp format), whereas 187 (63%) used iOS devices (m4a format). We found no difference in the distribution of age, gender, body mass index, smoking, antibiotic usage, and asthma, between the two types of devices (*P-value>.05*). The overall rate of comorbidities in this study was relatively low: there were 31 (10%) participants who used antibiotics and only 12 (4%) participants with asthma. More details are shown in Table 1.

For peer review only

For peer review only - http://bmjopen.bmj.com/site/about/guidelines.xhtml

Table 1: Study population characteristics

The clinical data in the table above describe the overall population of the study. The total number and its percentage are used to represent all categorical data. The table below summarizes general information for describing audio data.

All p-values comparing iOS (m4a) and Android users (3gp) were calculated using chi2 and Student's t-tests.

		All	m4a		3gp		P-values (m4a, 3gp)
			Female	Male	Female	Male	
**Participants (N)**	Total	296	107	80	51	58	-
**Age (years)**	mean (SD)	40.3 (12.6)	38.8 (13.4)	42.9 (12.7)	37.8 (11.6)	41.5 (11.3)	0.28
**Body Mass Index (kg/m²)**	mean (SD)	24.1 (4.7)	24.6 (5.5)	26.5 (4.1)	24.1 (3.8)	26.6 (4.17)	0.95
**Antibiotic (%)**	No	265 (90%)	93 (87%)	73 (91%)	44 (86%)	55 (95%)	0.87
	Yes	31 (10%)	14 (13%)	7 (9%)	7 (14%)	3 (5%)	
**Asthma (%)**	No	284 (96%)	104 (97%)	75 (94%)	47 (92%)	58 (100%)	0.82
	Yes	12 (4%)	3 (3%)	5 (6%)	4 (8%)	0 (0%)	
**Smoking (%)**	Never	199 (67%)	77 (72%)	51 (64%)	36 (71%)	35 (60%)	0.41
	Former smoker	53 (18%)	19 (18%)	20 (25%)	9 (18%)	13 (22%)	
	Current smoker	44 (15%)	11 (10%)	9 (11%)	6 (11%)	10 (18%)	
**Audio recordings**	Total	1772	584	499	345	344	<0.001
	No Fatigue	1222 (69%)	394 (67%)	370 (74%)	190 (55%)	268 (78%)	
	Fatigue	550 (31%)	190 (33%)	129 (26%)	155 (45%)	76 (22%)	
**Mean (SD) and maximum of audio recording per participant in the 14-day follow-up period**	mean (SD)	6 (5)	6 (5)	6 (5)	6 (5)	6 (5)	-
	max	16	14	16	15	14	

For peer review only - http://bmjopen.bmj.com/site/about/guidelines.xhtml

Participants reported their fatigue status on average 6 days during the first 14 days of follow-up, resulting in the analysis of 1772 audio recordings for each audio type (type 1 and type 2) when all inclusion criteria were met, including 550 audio recordings for participants with fatigue. In both audio sets, women reported experiencing fatigue at a higher rate than men (*P-value<.001*). Women constituted 155 (60%) of all fatigued Android users and 190 (67%) of all fatigued iOS users.

Prediction models

We reduced the extracted features from Mel-spectrograms to 250 top components with PCA, explaining 97% and 99% of the variance in the data for iOS and Android audio sets respectively. We then compared the performances of the machine learning algorithms to select the best models for the derivation of the vocal biomarkers.

The voting classifier was the best model selected for the development of the vocal biomarker for male iOS users, with an AUC of 82% and overall accuracy, precision, recall, and f1-score of 84%. The model selected for female iOS users was SVM with an overall precision of 80% and an AUC of 86%. For male Android users, the selected model is the voting classifier with a precision and recall of 89%, a f1-score of 88%, and a weighted AUC of 85%. For female Android users, the SVM was selected with an overall precision of 79% and an AUC of 79%. More details are shown in Table 2. The calibrations of the selected models were good (Mean Brier Scores = 0.15, 0.12, 0.17, and 0.12 respectively for Android female users, Android male users, iOS female users, and iOS male users).

Derivation of the digital fatigue vocal biomarker

Based on the model selected for each audio set, we derived the trained vocal biomarkers which quantitatively represent the probability of being labeled as fatigued. As shown in Figure 3, we found a significant difference in the distributions of vocal biomarkers between the fatigue and no fatigue classes in our testing dataset (t-test *P<.001*).

For peer review only

Table 2: Results of the prediction models

The selected models were selected using Recall_1 and weighted AUC and are highlighted in bold. Class 0: No fatigue, Class 1: Fatigue

Audio_format	Gender	ML model	Accuracy	Ov.Precision	Precision_0	Precision_1	Ov.Recall	Recall_0	Recall_1	Ov.f1score	f1-score_0	f1-score_1	Weighted AUC
3gp (Android)	Female	LR	0.77	0.77	0.81	0.73	0.77	0.76	0.77	0.77	0.78	0.75	0.85
		KNN	0.72	0.73	0.70	0.77	0.72	0.87	0.55	0.72	0.78	0.64	0.76
		**SVM**	**0.80**	**0.80**	**0.80**	**0.79**	**0.80**	**0.84**	**0.74**	**0.80**	**0.82**	**0.77**	**0.86**
		VC	0.78	0.78	0.81	0.75	0.78	0.79	0.77	0.78	0.80	0.76	0.86
	Male	LR	0.78	0.79	0.87	0.50	0.78	0.85	0.53	0.79	0.86	0.52	0.81
		KNN	0.83	0.83	0.83	0.80	0.83	0.98	0.27	0.79	0.90	0.40	0.84
		SVM	0.84	0.83	0.88	0.67	0.84	0.93	0.53	0.83	0.90	0.59	0.82
		**VC**	**0.84**	**0.84**	**0.89**	**0.64**	**0.84**	**0.91**	**0.60**	**0.84**	**0.90**	**0.62**	**0.82**
m4a (iOS)	Female	LR	0.72	0.72	0.80	0.56	0.72	0.77	0.61	0.72	0.79	0.58	0.75
		KNN	0.68	0.65	0.72	0.50	0.68	0.86	0.29	0.65	0.78	0.37	0.67
		**SVM**	**0.79**	**0.79**	**0.81**	**0.75**	**0.79**	**0.91**	**0.55**	**0.79**	**0.86**	**0.64**	**0.79**
		VC	0.77	0.76	0.80	0.69	0.77	0.89	0.53	0.76	0.84	0.60	0.78
	Male	LR	0.73	0.74	0.83	0.48	0.73	0.80	0.54	0.73	0.81	0.51	0.80
		KNN	0.89	0.89	0.89	0.89	0.89	0.97	0.65	0.88	0.93	0.76	0.81
		SVM	0.85	0.84	0.86	0.76	0.85	0.95	0.58	0.84	0.90	0.67	0.85
		**VC**	**0.89**	**0.89**	**0.89**	**0.89**	**0.89**	**0.97**	**0.65**	**0.88**	**0.93**	**0.76**	**0.85**

KNN: K-Nearest Neighbors, LR: Logistic Regression, Ov. : Overall, SVM: Support Vector Machine, VC: Voting Classifier

For peer review only

Discussion

In this study, we built an AI-based pipeline to develop a vocal biomarker for both genders and both types of smartphones (male/female, Android/iOS) that effectively recognize fatigued and non-fatigued participants with COVID-19.

We stratified the data to prevent data heterogeneity, which is considered contamination and makes it difficult to build a reliable and consistent classification model(s), resulting in poorer prediction performance. This contamination is caused by two factors: first, significant gender differences in fatigability, since it has previously been shown that men and women experience and report fatigue differently. And second, different microphone types incorporated in both smartphone devices used by the participants (iOS and Android), which have a direct impact on the quality of the recorded audios (machine learning algorithms separate the audio formats rather than the fatigue status if there is no constant microphone. (see Supplementary Online Material 2 for more details).

With the increased interest in remote voice analysis as a noninvasive and powerful telemedicine tool, various studies have been carried out, mostly in neurological disorders (eg, Parkinson's disease[11] and Alzheimer's disease[29]) and mental health (eg. stress and depression[30]). Recently, a significant research effort has evolved to employ respiratory sounds for COVID-19 and the main focus was on the use of cough[17,31] and breathing[32] to develop a COVID-19 screening tool. However, no previous work has been devoted to investigating the association of voice with COVID-19 symptoms, precisely fatigue.

Fatigue is one of the commonly reported symptoms of COVID-19 and Long-COVID syndrome[33], which can persist regardless of how severe COVID-19's acute stage is[34]. A variety of cerebral, peripheral, and psychosocial factors[35,7] play a role in the development of fatigue. It may also occur from chronic inflammation in the brain and at neuromuscular junctions. New evidence shows that patients with Long-COVID syndrome continue to have higher measures of blood clotting, thrombosis[36], which may also explain the persistence of fatigue. COVID-19 is associated with variations in airway resistance[37]. This narrowing of the airway is manifested in the increase in

For peer review only - http://bmjopen.bmj.com/site/about/guidelines.xhtml

audible turbulence in both sighing and yawning, which is frequently associated with fatigue[38].

Human voice is produced by the flow of air from the lungs through the larynx, which causes the vocal fold vibrations, generating a pulsating air stream[39]. The process is controlled by the laryngeal muscle activation[40] but involves the entire respiratory system to provide the air pressure necessary for phonation. Decreased pulmonary function in COVID-19 patients can cause reduced glottal airflow that is essential for normal voice production[41]. Furthermore, in case of increased fatigue, the voice production process may be additionally disturbed due to reduced laryngeal muscle tension, resulting in dysphonia that appears in up to 49% of COVID-19 patients[41].

Study Limitations

This study has several limitations. First, although our data was stratified based on gender and smartphone devices, the mix of languages might also result in different voice features subsequently, in different model performances. There is presently no comparable dataset with similar audio recordings for further external validation of our findings. Thus, more data should be collected to improve the transferability of our vocal biomarker to other populations. Second, our data labeling was only based on a qualitative self-reported fatigue status. A fatigue severity scale would allow a quantitative assessment of fatigue severity in a uniform and unbiased way throughout all participants. Finally, time series voice analysis for each participant was not included in the study. More investigation, including time series analysis, would establish a personalized baseline for each participant, potentially enhancing the performance of our vocal biomarkers.

Conclusion

In this study, we demonstrated the association between fatigue and voice in people with COVID-19 and developed a fatigue vocal biomarker that can accurately predict the presence of fatigue. These findings suggest that vocal biomarkers, digitally incorporated into telemonitoring technologies, might be used to identify and remotely monitor this symptom in patients suffering from COVID-19 as well as other chronic diseases.

For peer review only - http://bmjopen.bmj.com/site/about/guidelines.xhtml

Acknowledgments

We thank all participants that accepted to be involved in the study, members that collaborated to the launch and monitoring of the Predi-COVID cohort, as well as its scientific committee, the IT team responsible for the development of the application, and the nurses in charge of recruitment, data collection, and management on the field.

Contributors

Elbéji and Fagherazzi had full access to all of the data in the study and took responsibility for the integrity of the data and the accuracy of the data analysis.

Concept and design: Fagherazzi, Zhang, Fischer.

Acquisition, analysis, or interpretation of data: All authors.

Drafting of the manuscript: Elbéji.

Critical revision of the manuscript for important intellectual content: All authors.

Statistical analysis: Elbéji, Zhang, Higa, Fischer.

Obtained funding: Fagherazzi.

Administrative, technical, or material support: Fischer.

Funding and Conflict of Interests Statement

The Predi-COVID study is supported by the Luxembourg National Research Fund (FNR) (Predi-COVID, grant number 14716273), the André Losch Foundation, and the Luxembourg Institute of Health.

Competing interests: None declared.

For peer review only

Data Sharing Statement

Audio data, datasets and source code used in this study are publicly available.
Audio data available in Zenodo repository, [DOI: 10.5281/zenodo.5937844]
Datasets and source code available in Github, [https://github.com/LIHVOICE/Predi_COVID_Fatigue_Vocal_Biomarker].

Ethics Statement

The National Research Ethics Committee of Luxembourg (study number 202003/07) gave a favorable opinion to the study in April 2020.

For peer review only

References

1 WHO Coronavirus (COVID-19) Dashboard. https://covid19.who.int (accessed Aug 5, 2021).

2 Website. https://www.oecd.org/coronavirus/policy-responses/the-territorial-impact-of-covid-19-managing-the-crisis-and-recovery-across-levels-of-government-a2c6abaf/.

3 Qi R, Chen W, Liu S, *et al.* Psychological morbidities and fatigue in patients with confirmed COVID-19 during disease outbreak: prevalence and associated biopsychosocial risk factors. *medRxiv* 2020; : 2020.05.08.20031666.

4 Tansey CM, Louie M, Loeb M, *et al.* One-year outcomes and health care utilization in survivors of severe acute respiratory syndrome. *Arch Intern Med* 2007; **167**: 1312–20.

5 Neuropsychological and neurophysiological correlates of fatigue in post-acute patients with neurological manifestations of COVID-19: Insights into a challenging symptom. *J Neurol Sci* 2021; **420**: 117271.

6 Rudroff T, Fietsam AC, Deters JR, Bryant AD, Kamholz J. Post-COVID-19 Fatigue: Potential Contributing Factors. *Brain Sciences* 2020; **10**: 1012.

7 Morgul E, Bener A, Atak M, *et al.* COVID-19 pandemic and psychological fatigue in Turkey. International Journal of Social Psychiatry. 2021; **67**: 128–35.

For peer review only - http://bmjopen.bmj.com/site/about/guidelines.xhtml

8 Hunter SK. Sex differences in human fatigability: mechanisms and insight to physiological responses. *Acta Physiol* 2014; **210**: 768–89.

9 Gunasekeran DV, Tseng RMW, Tham Y-C, Wong TY. Applications of digital health for public health responses to COVID-19: a systematic scoping review of artificial intelligence, telehealth and related technologies. *NPJ Digital Medicine* 2021; **4**. DOI:10.1038/s41746-021-00412-9.

10 DeMerle K, Angus DC, Seymour CW. Precision Medicine for COVID-19: Phenotype Anarchy or Promise Realized? *JAMA* 2021; **325**: 2041–2.

11 Tracy JM, Özkanca Y, Atkins DC, Hosseini GR. Investigating voice as a biomarker: Deep phenotyping methods for early detection of Parkinson's disease. *J Biomed Inform* 2020; **104**. DOI:10.1016/j.jbi.2019.103362.

12 Laguarta J, Subirana B. Longitudinal Speech Biomarkers for Automated Alzheimer's Detection. Frontiers in Computer Science. 2021; **3**. DOI:10.3389/fcomp.2021.624694.

13 Cho S-W, Yin CS, Park Y-B, Park Y-J. Differences in self-rated, perceived, and acoustic voice qualities between high- and low-fatigue groups. *J Voice* 2011; **25**: 544–52.

14 Whitmore J, Fisher S. Speech during sustained operations. Speech Communication. 1996; **20**: 55–70.

15 Vollrath M. Automatic measurement of aspects of speech reflecting motor coordination. Behavior Research Methods, Instruments, & Computers. 1994; **26**: 35–40.

16 Greeley HP, Berg J, Friets E, *et al.* Fatigue estimation using voice analysis. Behavior Research Methods. 2007; **39**: 610–9.

17 Detection of COVID-19 from voice, cough and breathing patterns: Dataset and preliminary results. *Comput Biol Med* 2021; **138**: 104944.

18 Orlandic L, Teijeiro T, Atienza D. The COUGHVID crowdsourcing dataset, a corpus for the study of large-scale cough analysis algorithms. Scientific Data.

For peer review only - http://bmjopen.bmj.com/site/about/guidelines.xhtml

2021; **8**. DOI:10.1038/s41597-021-00937-4.

19 Bartl-Pokorny KD, Pokorny FB, Batliner A, *et al.* The voice of COVID-19: Acoustic correlates of infection in sustained vowels. *J Acoust Soc Am* 2021; **149**: 4377.

20 Fagherazzi G, Fischer A, Betsou F, *et al.* Protocol for a prospective, longitudinal cohort of people with COVID-19 and their household members to study factors associated with disease severity: the Predi-COVID study. *BMJ Open* 2020; **10**: e041834.

21 United Nations. Universal Declaration of Human Rights | United Nations. https://www.un.org/en/about-us/universal-declaration-of-human-rights (accessed Nov 18, 2021).

22 Hoffmann W, Latza U, Baumeister SE, *et al.* Guidelines and recommendations for ensuring Good Epidemiological Practice (GEP): a guideline developed by the German Society for Epidemiology. *Eur J Epidemiol* 2019; **34**: 301–17.

23 [datasets] LIHVOICE.Predi_COVID_Fatigue_Vocal_Biomarker/Android_audioset.csv at main · LIHVOICE/Predi_COVID_Fatigue_Vocal_Biomarker. GitHub. https://github.com/LIHVOICE/Predi_COVID_Fatigue_Vocal_Biomarker (accessed Jan 31, 2022).

24 [datasets] Elbéji A, Zhang L, Higa E, *et al.* Audio recordings of COVID-19 positive individuals from the prospective Predi-COVID cohort study with their fatigue status. 2022; published online Feb 1. DOI:10.5281/zenodo.5937844.

25 Barman R, Deshpande S, Agarwal S, Inamdar U, Devare M. Transfer Learning for Small Dataset. 2019; published online March 26. http://dx.doi.org/ (accessed Nov 18, 2021).

26 Weiss K, Khoshgoftaar TM, Wang D. A survey of transfer learning. *Journal of Big Data* 2016; **3**: 1–40.

27 Simonyan K, Zisserman A. Very Deep Convolutional Networks for Large-Scale Image Recognition. 2014; published online Sept 4. http://arxiv.org/abs/1409.1556

For peer review only - http://bmjopen.bmj.com/site/about/guidelines.xhtml

For peer review only

(accessed Aug 6, 2021).

28 Hasan BMS, Abdulazeez AM. A Review of Principal Component Analysis Algorithm for Dimensionality Reduction. *Journal of Soft Computing and Data Mining* 2021; **2**: 20–30.

29 König A, Satt A, Sorin A, *et al.* Automatic speech analysis for the assessment of patients with predementia and Alzheimer's disease. *Alzheimer's & dementia (Amsterdam, Netherlands)* 2015; **1**. DOI:10.1016/j.dadm.2014.11.012.

30 Zhang L, Duvvuri R, Chandra KKL, Nguyen T, Ghomi RH. Automated voice biomarkers for depression symptoms using an online cross-sectional data collection initiative. *Depress Anxiety* 2020; **37**. DOI:10.1002/da.23020.

31 Noninvasive Vocal Biomarker is Associated With Severe Acute Respiratory Syndrome Coronavirus 2 Infection. *Mayo Clinic Proceedings: Innovations, Quality & Outcomes* 2021; **5**: 654–62.

32 COVID-19 Sounds App. http://www.covid-19-sounds.org/ (accessed Nov 18, 2021).

33 Goërtz YMJ, Van Herck M, Delbressine JM, *et al.* Persistent symptoms 3 months after a SARS-CoV-2 infection: the post-COVID-19 syndrome? *ERJ Open Res* 2020; **6**. DOI:10.1183/23120541.00542-2020.

34 Townsend L, Dyer AH, Jones K, *et al.* Persistent fatigue following SARS-CoV-2 infection is common and independent of severity of initial infection. *PLoS One* 2020; **15**: e0240784.

35 Arnold P, Njemini R, Vantieghem S, *et al.* Peripheral muscle fatigue in hospitalised geriatric patients is associated with circulating markers of inflammation. Experimental Gerontology. 2017; **95**: 128–35.

36 Fogarty H, Townsend L, Morrin H, *et al.* Persistent endotheliopathy in the pathogenesis of long COVID syndrome. *J Thromb Haemost* 2021; **19**. DOI:10.1111/jth.15490.

37 Pan S-Y, Ding M, Huang J, Cai Y, Huang Y-Z. Airway resistance variation

For peer review only

correlates with prognosis of critically ill COVID-19 patients: A computational fluid dynamics study. *Comput Methods Programs Biomed* 2021; **208**: 106257.

38 Murry T. The voice and its disorders, 4th edition. By Margaret C. L. Greene, 446 pp, illus, J. B. Lippincott Co., Philadelphia, PA, 1980. $47.50. 1981. DOI:10.1002/HED.2890030517.

39 Zörner S, Kaltenbacher M, Döllinger M. Investigation of prescribed movement in fluid–structure interaction simulation for the human phonation process. Computers & Fluids. 2013; **86**: 133–40.

40 Yin J, Zhang Z. Laryngeal muscular control of vocal fold posturing: Numerical modeling and experimental validation. *J Acoust Soc Am* 2016; **140**: EL280.

41 Dassie-Leite AP, Gueths TP, Ribeiro VV, Pereira EC, Martins P do N, Daniel CR. Vocal Signs and Symptoms Related to COVID-19 and Risk Factors for their Persistence. *J Voice* 2021; published online Aug 11. DOI:10.1016/j.jvoice.2021.07.013.

Legends

Table 1: Study population characteristics

Table 2: Results of the prediction models

Figure 1. General Pipeline

Figure 2. VGG19 Feature Extraction

Figure 3. Derivation of the digital fatigue vocal biomarker for Android and iOS users.

SOM 1. Text to read

SOM 2. VGG19 extracted features from participants' audio recordings

For peer review only

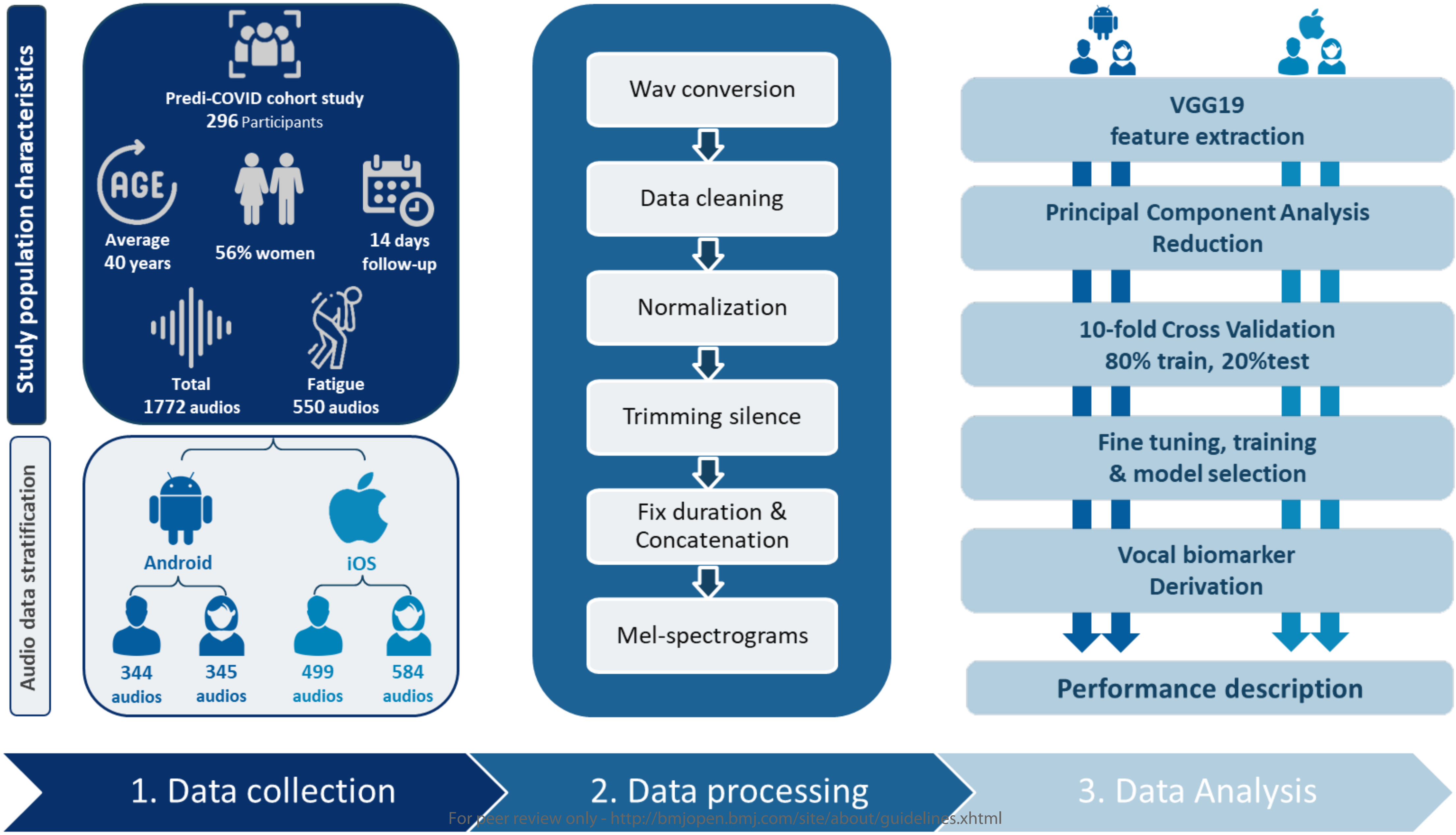

For peer review only - http://bmjopen.bmj.com/site/about/guidelines.xhtml

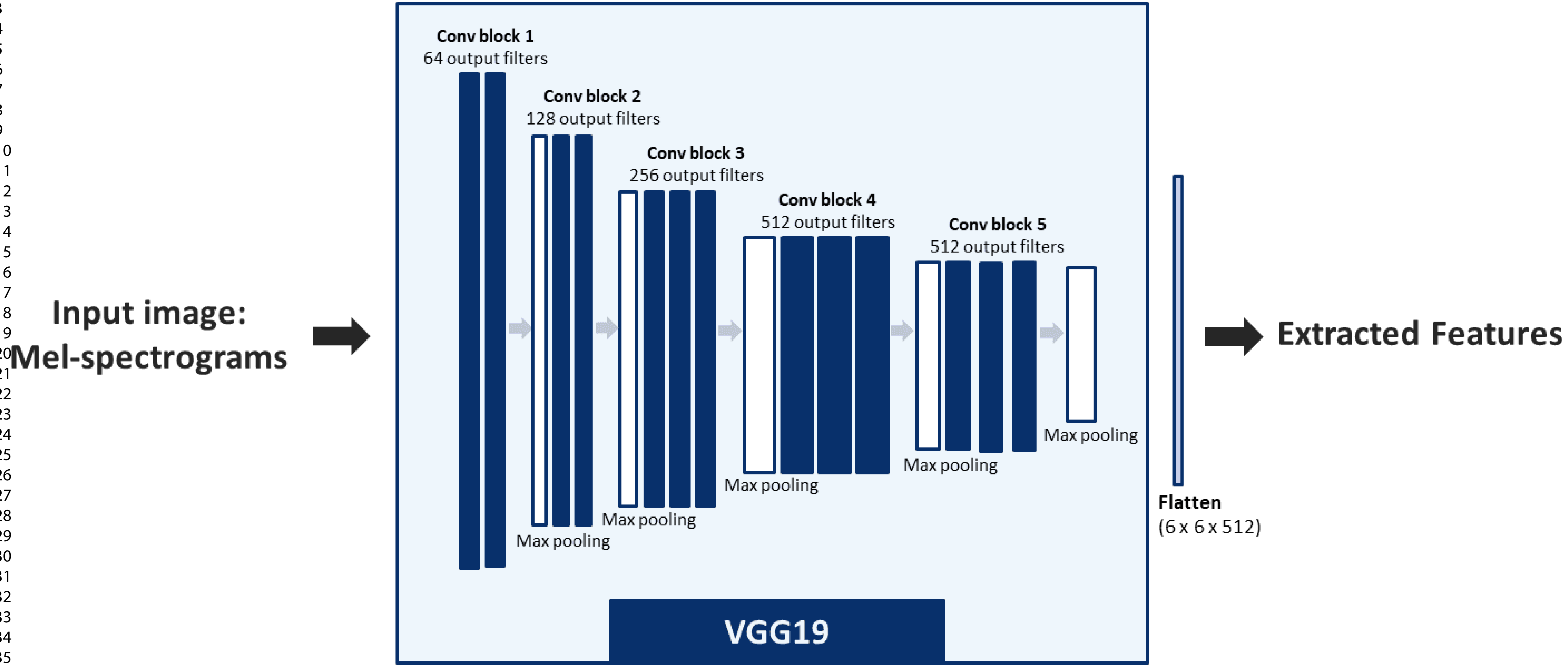
Input image:
Mel-spectrograms
Conv block 1
64 output filters
Conv block 2
128 output filters
Conv block 3
256 output filters
Conv block 4
512 output filters
Conv block 5
512 output filters
Max pooling
Max pooling
Max pooling
Max pooling
Max pooling
VGG19
Flatten
(6 x 6 x 512)
Extracted Features

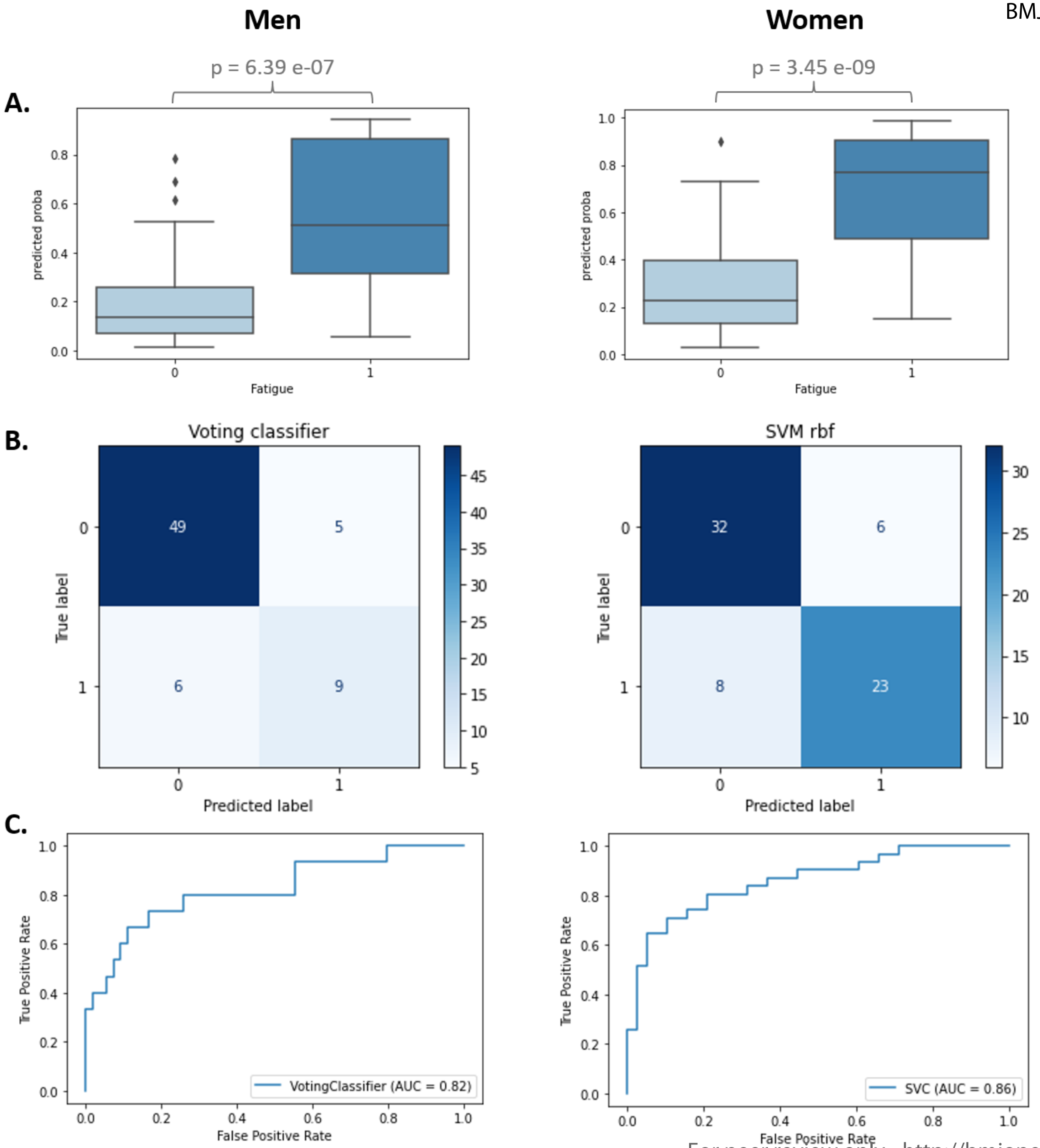

Figure 3a. Derivation of the digital fatigue vocal biomarker for Android users

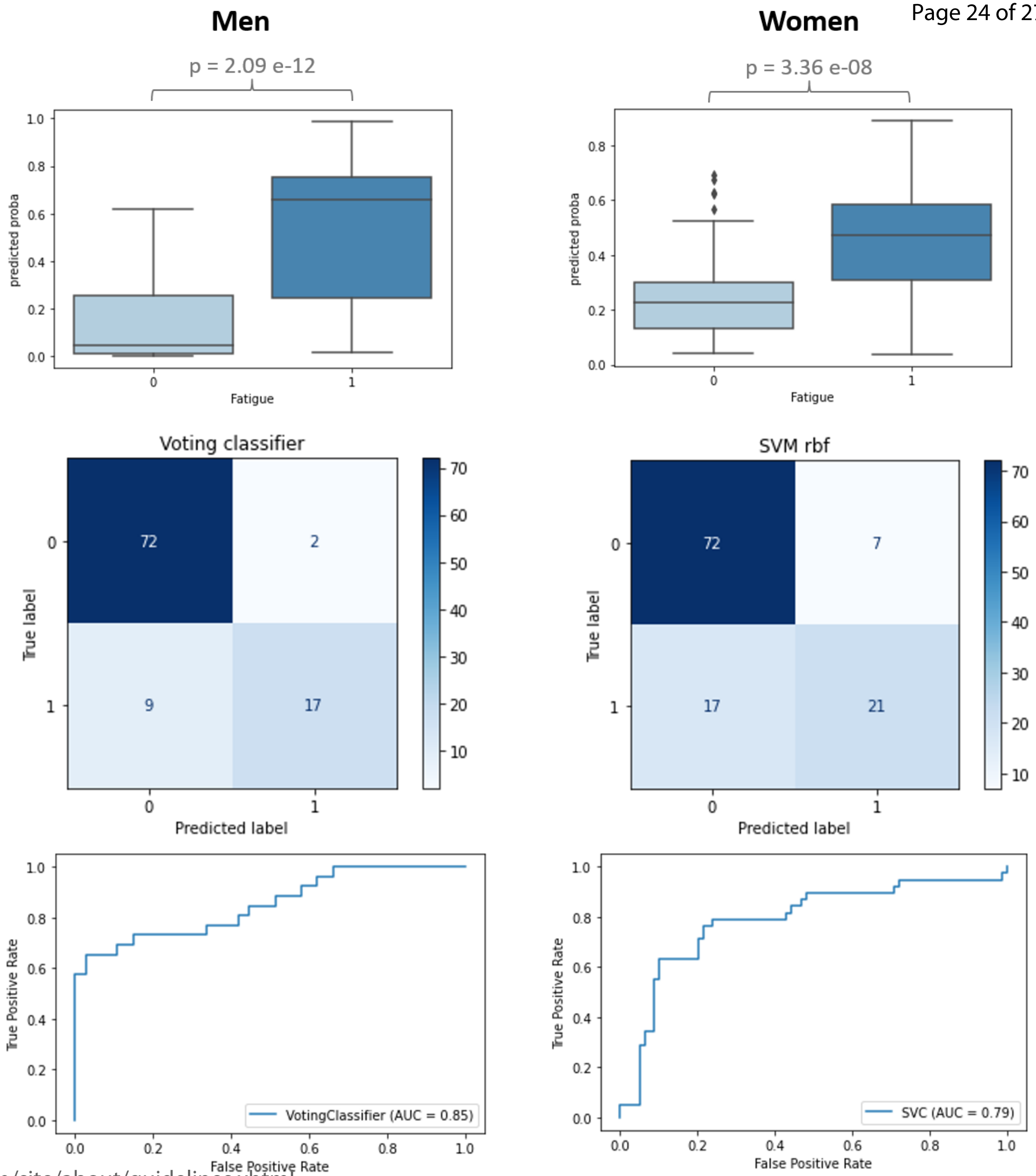

Figure 3b. Derivation of the digital fatigue vocal biomarker for iOS users

Supplementary Online Material 1. Standardized, prespecified text to be read by study participants to collect voice recordings.

Universal Declaration of Human Rights, United Nations.

English

Everyone has the right to a standard of living adequate for the health and well-being of himself and of his family, including food, clothing, housing and medical care and necessary social services, and the right to security in the event of unemployment, sickness, disability, widowhood, old age or other lack of livelihood in circumstances beyond his control.

French

Toute personne a droit à un niveau de vie suffisant pour assurer sa santé, son bien-être et ceux de sa famille, notamment pour l'alimentation, l'habillement, le logement, les soins médicaux ainsi que pour les services sociaux nécessaires ; elle a droit à la sécurité en cas de chômage, de maladie, d'invalidité, de veuvage, de vieillesse ou dans les autres cas de perte de ses moyens de subsistance par suite de circonstances indépendantes de sa volonté.

German

Jeder hat das Recht auf einen Lebensstandard, der seine und seiner Familie Gesundheit und Wohl gewährleistet, einschließlich Nahrung, Kleidung, Wohnung,

ärztliche Versorgung und notwendige soziale Leistungen gewährleistet sowie das Recht auf Sicherheit im Falle von Arbeitslosigkeit, Krankheit, Invalidität oder Verwitwung, im Alter sowie bei anderweitigem Verlust seiner Unterhaltsmittel durch unverschuldete Umstände.

Portuguese

Toda a pessoa tem direito a um nível de vida suficiente para lhe assegurar e à sua família a saúde e o bem-estar, principalmente quanto à alimentação, ao vestuário, ao alojamento, à assistência médica e ainda quanto aos serviços sociais necessários, e tem direito à segurança no desemprego, na doença, na invalidez, na viuvez, na velhice ou noutros casos de perda de meios de subsistência por circunstâncias independentes da sua vontade.

Supplementary Online Material 2. VGG19 extracted features from participants' audio recordings

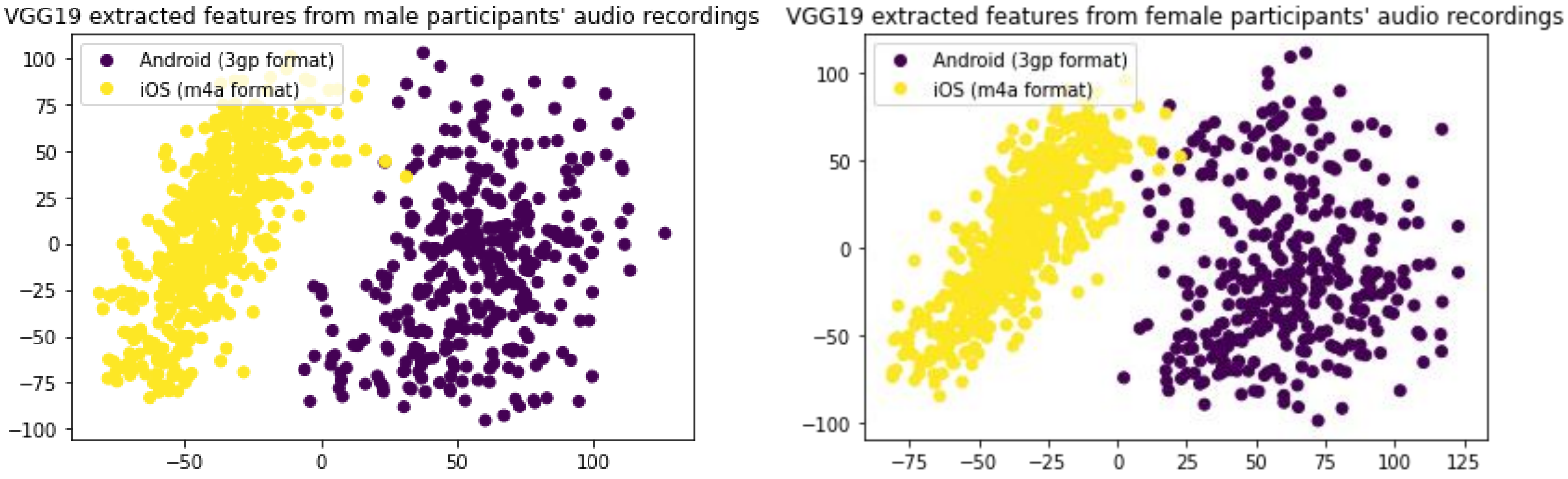

The scatter plot of the 250 relevant components given by PCA reduction revealed two distinct clusters. These two groups appeared to characterize the audio formats, m4a (iOS users) and 3gp (Android users).

It was consequently hypothesized that our data was heterogeneous and that it would be preferable to fit the models with each audio format independently.

TRIPOD Checklist: Prediction Model Development and Validation

Section/Topic	Item		Checklist Item	Page
**Title and abstract**				
Title	1	D;V	Identify the study as developing and/or validating a multivariable prediction model, the target population, and the outcome to be predicted.	1
Abstract	2	D;V	Provide a summary of objectives, study design, setting, participants, sample size, predictors, outcome, statistical analysis, results, and conclusions.	2
**Introduction**				
Background and objectives	3a	D;V	Explain the medical context (including whether diagnostic or prognostic) and rationale for developing or validating the multivariable prediction model, including references to existing models.	4
	3b	D;V	Specify the objectives, including whether the study describes the development or validation of the model or both.	5
**Methods**				
Source of data	4a	D;V	Describe the study design or source of data (e.g., randomized trial, cohort, or registry data), separately for the development and validation data sets, if applicable.	5
	4b	D;V	Specify the key study dates, including start of accrual; end of accrual; and, if applicable, end of follow-up.	
Participants	5a	D;V	Specify key elements of the study setting (e.g., primary care, secondary care, general population) including number and location of centres.	6
	5b	D;V	Describe eligibility criteria for participants.	
	5c	D;V	Give details of treatments received, if relevant.	
Outcome	6a	D;V	Clearly define the outcome that is predicted by the prediction model, including how and when assessed.	9
	6b	D;V	Report any actions to blind assessment of the outcome to be predicted.	
Predictors	7a	D;V	Clearly define all predictors used in developing or validating the multivariable prediction model, including how and when they were measured.	8
	7b	D;V	Report any actions to blind assessment of predictors for the outcome and other predictors.	
Sample size	8	D;V	Explain how the study size was arrived at.	7
Missing data	9	D;V	Describe how missing data were handled (e.g., complete-case analysis, single imputation, multiple imputation) with details of any imputation method.	
Statistical analysis methods	10a	D	Describe how predictors were handled in the analyses.	8
	10b	D	Specify type of model, all model-building procedures (including any predictor selection), and method for internal validation.	
	10c	V	For validation, describe how the predictions were calculated.	
	10d	D;V	Specify all measures used to assess model performance and, if relevant, to compare multiple models.	
	10e	V	Describe any model updating (e.g., recalibration) arising from the validation, if done.	
Risk groups	11	D;V	Provide details on how risk groups were created, if done.	
Development vs. validation	12	V	For validation, identify any differences from the development data in setting, eligibility criteria, outcome, and predictors.	9
**Results**				
Participants	13a	D;V	Describe the flow of participants through the study, including the number of participants with and without the outcome and, if applicable, a summary of the follow-up time. A diagram may be helpful.	9
	13b	D;V	Describe the characteristics of the participants (basic demographics, clinical features, available predictors), including the number of participants with missing data for predictors and outcome.	
	13c	V	For validation, show a comparison with the development data of the distribution of important variables (demographics, predictors and outcome).	
Model development	14a	D	Specify the number of participants and outcome events in each analysis.	9
	14b	D	If done, report the unadjusted association between each candidate predictor and outcome.	
Model specification	15a	D	Present the full prediction model to allow predictions for individuals (i.e., all regression coefficients, and model intercept or baseline survival at a given time point).	10
	15b	D	Explain how to the use the prediction model.	
Model performance	16	D;V	Report performance measures for the prediction model.	10
Model-updating	17	V	If done, report the results from any model updating (i.e., model specification, model performance).	
**Discussion**				
Limitations	18	D;V	Discuss any limitations of the study (such as nonrepresentative sample, few events per predictor, missing data).	11
Interpretation	19a	V	For validation, discuss the results with reference to performance in the development data, and any other validation data.	10
	19b	D;V	Give an overall interpretation of the results, considering objectives, limitations, results from similar studies, and other relevant evidence.	
Implications	20	D;V	Discuss the potential clinical use of the model and implications for future research.	12
**Other information**				
Supplementary information	21	D;V	Provide information about the availability of supplementary resources, such as study protocol, Web calculator, and data sets.	5,6,10
Funding	22	D;V	Give the source of funding and the role of the funders for the present study.	3

*Items relevant only to the development of a prediction model are denoted by D, items relating solely to a validation of a prediction model are denoted by V, and items relating to both are denoted D;V. We recommend using the TRIPOD Checklist in conjunction with the TRIPOD Explanation and Elaboration document.

BMJ Open

Vocal biomarker predicts fatigue in people with COVID-19: results from the prospective Predi-COVID cohort study

Journal:	*BMJ Open*
Manuscript ID	bmjopen-2022-062463.R1
Article Type:	Original research
Date Submitted by the Author:	22-Jul-2022
Complete List of Authors:	Elbéji, Abir; Luxembourg Institute of Health, Department of Precision Health<br>Zhang, Lu; Luxembourg Institute of Health, Department of Precision Health<br>Higa, Eduardo; Luxembourg Institute of Health, Department of Precision Health<br>Fischer, Aurélie; Luxembourg Institute of Health, Departement of Population Health<br>Despotovic, Vladimir; Luxembourg Institute of Health, Department of Precision Health<br>Nazarov, Petr V.; Luxembourg Institute of Health, Department of Precision Health<br>Aguayo, Gloria; Luxembourg Institute of Health, Department of Population Health<br>Fagherazzi, Guy; Luxembourg Institute of Health, Department of Precision Health
**Primary Subject Heading**:	Health informatics
Secondary Subject Heading:	Global health
Keywords:	COVID-19, Health informatics < BIOTECHNOLOGY & BIOINFORMATICS, Public health < INFECTIOUS DISEASES

SCHOLARONE™
Manuscripts

I, the Submitting Author has the right to grant and does grant on behalf of all authors of the Work (as defined in the below author licence), an exclusive licence and/or a non-exclusive licence for contributions from authors who are: i) UK Crown employees; ii) where BMJ has agreed a CC-BY licence shall apply, and/or iii) in accordance with the terms applicable for US Federal Government officers or employees acting as part of their official duties; on a worldwide, perpetual, irrevocable, royalty-free basis to BMJ Publishing Group Ltd ("BMJ") its licensees and where the relevant Journal is co-owned by BMJ to the co-owners of the Journal, to publish the Work in this journal and any other BMJ products and to exploit all rights, as set out in our licence.

The Submitting Author accepts and understands that any supply made under these terms is made by BMJ to the Submitting Author unless you are acting as an employee on behalf of your employer or a postgraduate student of an affiliated institution which is paying any applicable article publishing charge ("APC") for Open Access articles. Where the Submitting Author wishes to make the Work available on an Open Access basis (and intends to pay the relevant APC), the terms of reuse of such Open Access shall be governed by a Creative Commons licence – details of these licences and which Creative Commons licence will apply to this Work are set out in our licence referred to above.

Other than as permitted in any relevant BMJ Author's Self Archiving Policies, I confirm this Work has not been accepted for publication elsewhere, is not being considered for publication elsewhere and does not duplicate material already published. I confirm all authors consent to publication of this Work and authorise the granting of this licence.

For peer review only

Vocal biomarker predicts fatigue in people with COVID-19: results from the prospective Predi-COVID cohort study

Abir Elbéji[1], Lu Zhang[2], Eduardo Higa[1], Aurélie Fischer[1], Vladimir Despotovic[2], Petr V. Nazarov[2], Gloria A. Aguayo[1], Guy Fagherazzi[1]

1. Deep Digital Phenotyping Research Unit. Department of Precision Health. Luxembourg Institute of Health, 1 A-B rue Thomas Edison, L-1445 Strassen, Luxembourg

2. Bioinformatics Platform. Luxembourg Institute of Health, 1A-B, rue Thomas Edison, L-1445 Strassen, Luxembourg

Reprints and correspondence:

Dr Guy Fagherazzi, PhD, ADR

Director of Department of Precision Health

Head of the Deep Digital Phenotyping Research Unit

Luxembourg Institute of Health

1 AB rue Thomas Edison, L-1445 STRASSEN, LUXEMBOURG

Phone: +352 6 21 58 95 54

Email: guy.fagherazzi@lih.lu

Website: https://ddp.lih.lu/

For peer review only - http://bmjopen.bmj.com/site/about/guidelines.xhtml

Abstract

Objective

To develop a vocal biomarker for fatigue monitoring in people with COVID-19.

Design Prospective cohort study.

Setting Predi-COVID data between May 2020 and May 2021.

Participants

A total of 1772 voice recordings was used to train an AI-based algorithm to predict fatigue, stratified by gender and smartphone's operating system (Android/iOS). The recordings were collected from 296 participants tracked for two weeks following SARS-CoV-2 infection.

primary and secondary outcome measures

Four machine learning algorithms (Logistic regression, k-nearest neighbors, support vector machine, and soft voting classifier) were used to train and derive the fatigue vocal biomarker. A t-test was used to evaluate the distribution of the vocal biomarker between the two classes (Fatigue and No fatigue).

Results

The final study population included 56% of women and had a mean (±SD) age of 40 (±13) years. Women were more likely to report fatigue (*P<.001*). We developed four models for Android female, Android male, iOS female, and iOS male users with a weighted AUC of 86%, 82%, 79%, 85%, and a mean Brier Score of 0.15, 0.12, 0.17, 0.12, respectively. The vocal biomarker derived from the prediction models successfully discriminated COVID-19 participants with and without fatigue .

Conclusions

This study demonstrates the feasibility of identifying and remotely monitoring fatigue thanks to voice. Vocal biomarkers, digitally integrated into telemedicine technologies, are expected to improve the monitoring of people with COVID-19 or Long-COVID.

ClinicalTrials.gov Identifier: NCT04380987

For peer review only

Strengths and limitations

-This is the first study supporting the hypothesis that fatigue can be accurately monitored based on voice in people with COVID-19.

-The analyses were based on a multi-lingual database of standardized voice recordings collected in real-life from people with confirmed SARS-CoV-2 infection as determined by PCR.

-There is no similar dataset available yet in the literature to replicate our findings.

-The vocal biomarker is trained on a binary outcome (Fatigue, Yes/No) and does not reflect the entire spectrum of fatigue severity. Further work should be performed in that direction.

For peer review only

Introduction

Coronavirus disease 2019 (COVID-19) is a global outbreak. More than 199 million confirmed cases of COVID-19 have been detected worldwide as of 4 August 2021, with more than 4 million deaths reported by the World Health Organization[1]. The worldwide population and healthcare systems have been greatly impacted by the COVID-19 pandemic. The pandemic has essentially put whole healthcare systems under pressure, requiring national or regional lockdowns[2]. Finding solutions that allow healthcare providers to focus on the more important and urgent patients, was, and still is, critical.

This outbreak continues to impact people, with many patients suffering from a range of acute symptoms, such as fatigue. Fatigue is a common symptom in patients with COVID-19 that can impact their quality of life, treatment adherence, and can be associated with numerous complications[3]. Recent findings showed that fatigue is a major symptom of the frequently reported Long-COVID syndrome. After recovering from the acute disease caused by the SARS outbreak, up to 60% of patients reported chronic fatigue 12 months later[4]. This supports the need for long-term monitoring solutions for these patients.

In general, fatigue can be of two types: physical and mental[5] experiencing lack of energy, inability to start and perform everyday activities, and lack of desire to do things. In the context of COVID-19, determinants of fatigue were categorized as both central and psychological factors, the latest might also be indirectly caused by pandemic-related fear and anxiety[6,7].

Fatigue affects men and women differently and has previously been shown to be reported differently in the two genders. Men and women have different anatomy and physiology, resulting in significant sex differences in fatigability[8].

Telemedicine, artificial intelligence (AI), and big data predictive analytics are examples of digital health technologies that have the potential to minimize the damaging effects of COVID-19 by improving responses to public health problems at a population level[9]. Using telemonitoring technologies to enable self-surveillance and remote monitoring of symptoms might therefore help to improve and personalize COVID-19 care delivery[10].

For peer review only

Voice is a promising source of digital data since it is rich, user-friendly, inexpensive to collect, and non-invasive, and can be used to develop vocal biomarkers that characterize disease states. Previous research was mostly conducted in the field of neurodegenerative diseases, such as Parkinson's disease[11] and Alzheimer's disease[12]. There are also studies that confirm the relation of voice disorders to fatigue, e.g., in Chronic Fatigue Syndrome (CFS). Neuromuscular, neuropsychological and hormonal dysfunction associated with CFS can influence the phonation and articulation, and alter tension, viscosity and thickness of the tissue of the larynx, tongue and lips, leading to decreased voice quality[13]. Increased fatigue affects voice characteristics, such as pitch, word duration[14] and timing of articulated sounds[15]. Vocal changes related to fatigue are more observed in consonant sounds that require a high average airflow[16].

In the context of the COVID-19 pandemic, respiratory sounds (e.g coughs, breathing, and voice) are also used as sources of information to develop COVID-19 screening tools[17,18,19]. However, no previous work has been devoted to investigating the association of voice with COVID-19 symptoms.

We hypothesized that there is an association between fatigue and voice in patients with COVID-19 and that it is possible to train an AI-based model to identify fatigue and subsequently generate a digital vocal biomarker for fatigue monitoring. We used data from the large hybrid prospective Predi-COVID cohort study to investigate this hypothesis.

Methods

Study design

This project uses data from the Predi-COVID study[20]. Predi-COVID is a hybrid cohort study that started in May 2020 in Luxembourg and involved participants who should meet all of the following requirements: (1) a signed informed consent form; (2) participants with confirmed SARS-CoV-2 infection as determined by PCR at one of Luxembourg's certified laboratories; and (3) 18 years and older.

This study combines data from the national surveillance system, which is used for virtually all COVID-19 positive patients. Biological sampling, electronic patient-

For peer review only - http://bmjopen.bmj.com/site/about/guidelines.xhtml

reported outcomes, and smartphone voice recording were collected to identify vocal biomarkers of respiratory syndromes and fatigue in this study. More details about the Predi-COVID study can be found elsewhere[20].

Health Inspection collaborators made the initial phone contact with potential participants. Those who consented to participate were contacted by a qualified nurse from the Clinical and Epidemiological Investigation Center (CIEC - Luxembourg Institute of Health), who outlined the study and arranged home or hospital visits.

Patient and Public Involvement

The Predi-COVID initiative was an emergency response from national research institutions grouped under 'Research Luxembourg' to fight the COVID-19 pandemic in Luxembourg and contribute to the general effort in the crisis. Therefore, for timing and safety reasons, patients with COVID-19 were not directly included to participate in the study design. However, the first participants included in Predi-COVID provided feedback on general workflow, data collection, questionnaires, and sampling, which was taken into account in an amendment to the protocol[20].

Data collection

Participants were followed for up to a year using a smartphone app to collect voice data. To ensure a minimum quality level, participants were asked to record it in a quiet environment while maintaining a certain distance from the microphone, and an audio example of what was required was also provided.

All the participants of this study were invited to record two audio types. The first, Type 1 audio, required participants to read paragraph 1 of article 25 of the Declaration of Human Rights[21], in their preferred language: French, German, English, or Portuguese; and the second, Type 2 audio, required them to hold the [a] vowel phonation without breathing for as long as they could (see Supplementary Online Material 1 for more details).

Predi-COVID collects data in conformity with the German Society of Epidemiology's best practices guidelines[22]. To draft the manuscript, we followed the TRIPOD criteria

For peer review only - http://bmjopen.bmj.com/site/about/guidelines.xhtml

for reporting AI-based model development and validation, as well as the corresponding checklist.

All Predi-COVID participants recruited between May 2020 and May 2021 who reported their fatigue status ("I feel well" as "No Fatigue" and "I am fatigued"/"I don't feel well" as "Fatigue") on the same day as the audio recordings during the 14 days of follow-up were included in this study[23]. As a result, several audio recordings for a single participant were available for both audio types[24].

Audio characteristics and vocal biomarker training

The audio recordings were collected in two formats, 3gp format (Android devices) and m4a format (iOS devices). Based on the smartphone's operating system and the user's gender (male/female), we trained one model for each category. This stratification was performed to minimize data heterogeneity and deal with sex as a potential confounding bias.

Audio pre-processing

All of the raw audio recordings were pre-processed (Figure 1). They were initially converted to .wav files, with audios lasting less than 2 seconds being excluded. Then, an audio clustering (DBSCAN) on basic features (duration, average, sum, and standard deviation of signal power, and fundamental frequency) was performed to detect outliers that were manually checked while excluding poor quality audios with 1) too noisy, 2) incorrect text reading, 3) type 1 and type 2 audios mixed, or 4) extended silence in the middle**.** Finally, peak normalization was used to boost the volume of quiet audio segments, and leading and trailing silences longer than 350 milliseconds were trimmed.

Feature extraction

We used transfer learning for the feature extraction process since it is adapted for small training databases[25]. Transfer learning is a technique where a model is constructed and trained with a set containing a large amount of data and then transfer and apply this learning to our dataset on top of it. It has the advantage of reducing the amount of data required while shortening training time and improving performance when compared to models built from scratch[26].

For peer review only - http://bmjopen.bmj.com/site/about/guidelines.xhtml

Convolutional neural networks require a fixed input size, whereas audio instances in our dataset were of variable length. To deal with this issue, Zero-padding was used to set the duration of each audio file to 50 seconds (the maximum length in our database). To raise the amount of information fed to the classifiers, type 1 (text reading) and type 2 ([a] phonation) audios were concatenated and used as a single input to the learning models.

All the audio recordings were first resampled to 8kHz and then converted to Mel-spectrograms using the Librosa library in Python. The hop-length was 2048 samples, and the number of Mel coefficients was set to 196. The Mel spectrograms were passed through VGG19 convolutional neural network architecture provided by Keras, which was pre-trained on the ImageNet database[27]. This approach, presented in Figure 2, may be considered as a feature extraction step, as it converts audio recordings to 512 feature maps, each of a size 6x6, leading to a total of 18432 features.

This large number of features is computationally expensive. Principal Component Analysis (PCA)[28] is therefore used for dimensionality reduction and to select the number of relevant components explaining the maximum of the variance in the data.

For peer review only - http://bmjopen.bmj.com/site/about/guidelines.xhtml

Statistical analysis

We divided our data into “Fatigue” and “No Fatigue” groups based on the participant’s reported answers for the inclusion and daily fatigue assessment of Predi-COVID. To characterize participants, descriptive statistics were used, which included means, standard deviations for quantitative variables, and counts and percentages for qualitative variables. The two population groups (3gp (Android users) and m4a (iOS users)) were compared using a student test for continuous variables, and a $\chi 2$ test for categorical variables.

A 10-fold cross-validation procedure was conducted on the training cohort participants to evaluate four classification models (logistic regression (LR), k-nearest neighbors (KNN), support vector machine (SVM), and soft voting classifier (VC), scikit-learn implementation in Python) at different regularization levels via a grid search, with the following evaluation metrics: area under the ROC curve (AUC), accuracy, F1-score, precision, and recall. The Brier score was also used to evaluate the calibration of the selected models.

The predicted probability of being classified as fatigued from the best model was considered as our final vocal biomarker, which may be used as a quantitative metric to monitor fatigue.

We evaluated the vocal biomarker’s distribution in both classes (Fatigue and No Fatigue) and performed a t-test between the two groups.

Results

Study population characteristics

The final study population is composed of 296 participants of whom 165 were women (56%), with an average age of 40 years (SD = 13). To record both audio types,109 (37%) participants utilized Android smartphones (3gp format), whereas 187 (63%) used iOS devices (m4a format). We found no difference in the distribution of age, gender, body mass index, smoking, antibiotic usage, and asthma, between the two types of devices (*P-value>.05*). The overall rate of comorbidities in this study was relatively low: there were 31 (10%) participants who used antibiotics and only 12 (4%) participants with asthma. More details are shown in Table 1.

For peer review only

For peer review only - http://bmjopen.bmj.com/site/about/guidelines.xhtml

For peer review only

Table 1: Study population characteristics

The clinical data in the table above describe the overall population of the study. The total number and its percentage are used to represent all categorical data. The table below summarizes general information for describing audio data.

All p-values comparing iOS (m4a) and Andoid users (3gp) were calculated using chi2

For peer review only - http://bmjopen.bmj.com/site/about/guidelines.xhtml

and Student's t-tests.

		All	m4a		3gp		P-values (m4a, 3gp)
			Female	Male	Female	Male	
**Participants (N)**	Total	296	107	80	51	58	-
**Age (years)**	mean (SD)	40.3 (12.6)	38.8 (13.4)	42.9 (12.7)	37.8 (11.6)	41.5 (11.3)	0.28
**Body Mass Index (kg/m²)**	mean (SD)	24.1 (4.7)	24.6 (5.5)	26.5 (4.1)	24.1 (3.8)	26.6 (4.17)	0.95
**Antibiotic (%)**	No	265 (90%)	93 (87%)	73 (91%)	44 (86%)	55 (95%)	0.87
	Yes	31 (10%)	14 (13%)	7 (9%)	7 (14%)	3 (5%)	
**Asthma (%)**	No	284 (96%)	104 (97%)	75 (94%)	47 (92%)	58 (100%)	0.82
	Yes	12 (4%)	3 (3%)	5 (6%)	4 (8%)	0 (0%)	
**Smoking (%)**	Never	199 (67%)	77 (72%)	51 (64%)	36 (71%)	35 (60%)	0.41
	Former smoker	53 (18%)	19 (18%)	20 (25%)	9 (18%)	13 (22%)	
	Current smoker	44 (15%)	11 (10%)	9 (11%)	6 (11%)	10 (18%)	
**Audio recordings**	Total	1772	584	499	345	344	<0.001
	No Fatigue	1222 (69%)	394 (67%)	370 (74%)	190 (55%)	268 (78%)	
	Fatigue	550 (31%)	190 (33%)	129 (26%)	155 (45%)	76 (22%)	
**Mean (SD) and maximum of audio recording per participant in the 14-day follow-up period**	mean (SD)	6 (5)	6 (5)	6 (5)	6 (5)	6 (5)	-
	max	16	14	16	15	14	

Participants reported their fatigue status on average 6 days during the first 14 days of follow-up, resulting in the analysis of 1772 audio recordings for each audio type (type 1 and type 2) when all inclusion criteria were met, including 550 audio recordings for participants with fatigue. In both audio sets, women reported experiencing fatigue at a higher rate than men (*P-value<.001*). Women constituted 155 (60%) of all fatigued Android users and 190 (67%) of all fatigued iOS users.

For peer review only - http://bmjopen.bmj.com/site/about/guidelines.xhtml

Prediction models

We reduced the extracted features from Mel-spectrograms to 250 top components with PCA, explaining 97% and 99% of the variance in the data for iOS and Android audio sets respectively. We then compared the performances of the machine learning algorithms to select the best models for the derivation of the vocal biomarkers.

The voting classifier was the best model selected for the development of the vocal biomarker for male iOS users, with an AUC of 85% and overall accuracy, precision, recall, and f1-score of 89%. The model selected for female iOS users was SVM with an overall precision of 79% and an AUC of 79%. For male Android users, the selected model is the voting classifier with a precision, recall and f1-score of 84%, and a weighted AUC of 82%. For female Android users, the SVM was selected with an overall precision of 80% and an AUC of 86%. More details are shown in Table 2. The calibrations of the selected models were good (Mean Brier Scores = 0.15, 0.12, 0.17, and 0.12 respectively for Android female users, Android male users, iOS female users, and iOS male users).

Derivation of the digital fatigue vocal biomarker

Based on the model selected for each audio set, we derived the trained vocal biomarkers which quantitatively represent the probability of being labeled as fatigued. As shown in Figure 3, we found a significant difference in the distributions of vocal biomarkers between the fatigue and no fatigue classes in our testing dataset.

For peer review only

Table 2: Results of the prediction models

The selected models were selected using Recall_1 and weighted AUC and are highlighted in bold. Class 0: No fatigue, Class 1: Fatigue

Audio_format	Gender	ML model	Accuracy	Ov.Precision	Precision_0	Precision_1	Ov.Recall	Recall_0	Recall_1	Ov.f1score	f1-score_0	f1-score_1	Weighted AUC
3gp (Android)	Female	LR	0.77	0.77	0.81	0.73	0.77	0.76	0.77	0.77	0.78	0.75	0.85
		KNN	0.72	0.73	0.70	0.77	0.72	0.87	0.55	0.72	0.78	0.64	0.76
		**SVM**	**0.80**	**0.80**	**0.80**	**0.79**	**0.80**	**0.84**	**0.74**	**0.80**	**0.82**	**0.77**	**0.86**
		VC	0.78	0.78	0.81	0.75	0.78	0.79	0.77	0.78	0.80	0.76	0.86
	Male	LR	0.78	0.79	0.87	0.50	0.78	0.85	0.53	0.79	0.86	0.52	0.81
		KNN	0.83	0.83	0.83	0.80	0.83	0.98	0.27	0.79	0.90	0.40	0.84
		SVM	0.84	0.83	0.88	0.67	0.84	0.93	0.53	0.83	0.90	0.59	0.82
		**VC**	**0.84**	**0.84**	**0.89**	**0.64**	**0.84**	**0.91**	**0.60**	**0.84**	**0.90**	**0.62**	**0.82**
m4a (iOS)	Female	LR	0.72	0.72	0.80	0.56	0.72	0.77	0.61	0.72	0.79	0.58	0.75
		KNN	0.68	0.65	0.72	0.50	0.68	0.86	0.29	0.65	0.78	0.37	0.67
		**SVM**	**0.79**	**0.79**	**0.81**	**0.75**	**0.79**	**0.91**	**0.55**	**0.79**	**0.86**	**0.64**	**0.79**
		VC	0.77	0.76	0.80	0.69	0.77	0.89	0.53	0.76	0.84	0.60	0.78
	Male	LR	0.73	0.74	0.83	0.48	0.73	0.80	0.54	0.73	0.81	0.51	0.80
		KNN	0.89	0.89	0.89	0.89	0.89	0.97	0.65	0.88	0.93	0.76	0.81
		SVM	0.85	0.84	0.86	0.76	0.85	0.95	0.58	0.84	0.90	0.67	0.85
		**VC**	**0.89**	**0.89**	**0.89**	**0.89**	**0.89**	**0.97**	**0.65**	**0.88**	**0.93**	**0.76**	**0.85**

KNN: K-Nearest Neighbors, LR: Logistic Regression, Ov. : Overall, SVM: Support Vector Machine, VC: Voting Classifier

For peer review only

Discussion

In this study, we built an AI-based pipeline to develop a vocal biomarker for both genders and both types of smartphones (male/female, Android/iOS) that effectively recognize fatigued and non-fatigued participants with COVID-19.

We stratified the data to prevent data heterogeneity, which is considered contamination and makes it difficult to build a reliable and consistent classification model(s), resulting in poorer prediction performance. This contamination is caused by two factors: first, significant gender differences in fatigability, since it has previously been shown that men and women experience and report fatigue differently, and second, different microphone types incorporated in both smartphone devices used by the participants (iOS and Android), which have a direct impact on the quality of the recorded audios (machine learning algorithms separate the audio formats rather than the fatigue status if there is no constant microphone. (see Supplementary Online Material 2 for more details).

With the increased interest in remote voice analysis as a noninvasive and powerful telemedicine tool, various studies have been carried out, mostly in neurological disorders (eg, Parkinson's disease[11] and Alzheimer's disease[29]) and mental health (eg. stress and depression[30]). Recently, a significant research effort has evolved to employ respiratory sounds for COVID-19 and the main focus was on the use of cough[17,31] and breathing[32] to develop a COVID-19 screening tool. However, no previous work has been devoted to investigating the association of voice with COVID-19 symptoms, precisely fatigue.

Fatigue is one of the commonly reported symptoms of COVID-19 and Long-COVID syndrome[33], which can persist regardless of how severe COVID-19's acute stage is[34]. A variety of cerebral, peripheral, and psychosocial factors[35,7] play a role in the development of fatigue. It may also occur from chronic inflammation in the brain and at neuromuscular junctions. New evidence shows that patients with Long-COVID syndrome continue to have higher measures of blood clotting, thrombosis[36], which may also explain the persistence of fatigue. COVID-19 is associated with variations in airway resistance[37]. This narrowing of the airway is manifested in the increase in

For peer review only - http://bmjopen.bmj.com/site/about/guidelines.xhtml

audible turbulence in both sighing and yawning, which is frequently associated with fatigue[38].

Human voice is produced by the flow of air from the lungs through the larynx, which causes the vocal fold vibrations, generating a pulsating air stream[39]. The process is controlled by the laryngeal muscle activation[40] but involves the entire respiratory system to provide the air pressure necessary for phonation. Decreased pulmonary function in COVID-19 patients can cause reduced glottal airflow that is essential for normal voice production[41]. Furthermore, in case of increased fatigue, the voice production process may be additionally disturbed due to reduced laryngeal muscle tension, resulting in dysphonia that appears in up to 49% of COVID-19 patients[41].

Study Limitations

This study has several limitations. First, although our data was stratified based on gender and smartphone devices, the mix of languages might also result in different voice features subsequently, in different model performances. There is presently no comparable dataset with similar audio recordings for further external validation of our findings. Thus, more data should be collected to improve the transferability of our vocal biomarker to other populations. Second, our data labeling was only based on a qualitative self-reported fatigue status. A fatigue severity scale would allow a quantitative assessment of fatigue severity in a uniform and unbiased way throughout all participants. Finally, time series voice analysis for each participant was not included in the study. More investigation, including time series analysis, would establish a personalized baseline for each participant, potentially enhancing the performance of our vocal biomarkers.

Conclusion

In this study, we demonstrated the association between fatigue and voice in people with COVID-19 and developed a fatigue vocal biomarker that can accurately predict the presence of fatigue. These findings suggest that vocal biomarkers, digitally incorporated into telemonitoring technologies, might be used to identify and remotely monitor this symptom in patients suffering from COVID-19 as well as other chronic diseases.

For peer review only - http://bmjopen.bmj.com/site/about/guidelines.xhtml

Acknowledgments

We thank all participants that accepted to be involved in the study, members that collaborated to the launch and monitoring of the Predi-COVID cohort, as well as its scientific committee, the IT team responsible for the development of the application, and the nurses in charge of recruitment, data collection, and management on the field.

Contributors

Elbéji and Fagherazzi had full access to all of the data in the study and took responsibility for the integrity of the data and the accuracy of the data analysis.

Fagherazzi, Zhang, and Fischer conceptualized and designed the study.

Elbéji, Zhang, Higa, Fischer, Despotovic, Nazarov, Aguayo, and Fagherazzi collected and analyzed data and contributed to the interpretation.

The statistical analysis was carried out by Elbéji, Zhang, Higa, and Fischer.

Elbéji drafted the initial manuscript. Elbéji, Zhang, Higa, Fischer, Despotovic, Nazarov, Aguayo, and Fagherazzi critically revised the manuscript for more important intellectual content. Fagherazzi obtained the funding. Fischer provided administrative, technical, and material support. The corresponding author certifies that all listed authors fulfill the authorship criteria and that no other authors that meet the criteria have been omitted.

Funding Statement

The Predi-COVID study is supported by the Luxembourg National Research Fund (FNR) (Predi-COVID, grant number 14716273), the André Losch Foundation, and the Luxembourg Institute of Health.

Competing interests: None declared.

For peer review only

Data Sharing Statement

Audio data, datasets and source code used in this study are publicly available.

Audio data available in Zenodo repository, [DOI: 10.5281/zenodo.5937844]

Datasets and source code available in Github, [https://github.com/LIHVOICE/Predi_COVID_Fatigue_Vocal_Biomarker].

Ethics Statement

The National Research Ethics Committee of Luxembourg (study number 202003/07) gave a favorable opinion to the study in April 2020.

For peer review only

References

1 WHO Coronavirus (COVID-19) Dashboard. https://covid19.who.int (accessed Aug 5, 2021).

2 Website. https://www.oecd.org/coronavirus/policy-responses/the-territorial-impact-of-covid-19-managing-the-crisis-and-recovery-across-levels-of-government-a2c6abaf/.

3 Qi R, Chen W, Liu S, *et al.* Psychological morbidities and fatigue in patients with confirmed COVID-19 during disease outbreak: prevalence and associated biopsychosocial risk factors. *medRxiv* 2020; : 2020.05.08.20031666.

4 Tansey CM, Louie M, Loeb M, *et al.* One-year outcomes and health care utilization in survivors of severe acute respiratory syndrome. *Arch Intern Med* 2007; **167**: 1312–20.

5 Neuropsychological and neurophysiological correlates of fatigue in post-acute patients with neurological manifestations of COVID-19: Insights into a challenging symptom. *J Neurol Sci* 2021; **420**: 117271.

6 Rudroff T, Fietsam AC, Deters JR, Bryant AD, Kamholz J. Post-COVID-19 Fatigue: Potential Contributing Factors. *Brain Sciences* 2020; **10**: 1012.

7 Morgul E, Bener A, Atak M, *et al.* COVID-19 pandemic and psychological fatigue in Turkey. International Journal of Social Psychiatry. 2021; **67**: 128–35.

8 Hunter SK. Sex differences in human fatigability: mechanisms and insight to physiological responses. *Acta Physiol* 2014; **210**: 768–89.

9 Gunasekeran DV, Tseng RMW, Tham Y-C, Wong TY. Applications of digital health for public health responses to COVID-19: a systematic scoping review of artificial intelligence, telehealth and related technologies. *NPJ Digital Medicine* 2021; **4**. DOI:10.1038/s41746-021-00412-9.

10 DeMerle K, Angus DC, Seymour CW. Precision Medicine for COVID-19: Phenotype Anarchy or Promise Realized? *JAMA* 2021; **325**: 2041–2.

11 Tracy JM, Özkanca Y, Atkins DC, Hosseini GR. Investigating voice as a biomarker: Deep phenotyping methods for early detection of Parkinson's disease. *J Biomed Inform* 2020; **104**. DOI:10.1016/j.jbi.2019.103362.

12 Laguarta J, Subirana B. Longitudinal Speech Biomarkers for Automated Alzheimer's Detection. Frontiers in Computer Science. 2021; **3**. DOI:10.3389/fcomp.2021.624694.

13 Cho S-W, Yin CS, Park Y-B, Park Y-J. Differences in self-rated, perceived, and acoustic voice qualities between high- and low-fatigue groups. *J Voice* 2011; **25**: 544–52.

14 Whitmore J, Fisher S. Speech during sustained operations. Speech Communication. 1996; **20**: 55–70.

15 Vollrath M. Automatic measurement of aspects of speech reflecting motor coordination. Behavior Research Methods, Instruments, & Computers. 1994; **26**: 35–40.

16 Greeley HP, Berg J, Friets E, *et al.* Fatigue estimation using voice analysis. Behavior Research Methods. 2007; **39**: 610–9.

17 Detection of COVID-19 from voice, cough and breathing patterns: Dataset and preliminary results. *Comput Biol Med* 2021; **138**: 104944.

18 Orlandic L, Teijeiro T, Atienza D. The COUGHVID crowdsourcing dataset, a corpus for the study of large-scale cough analysis algorithms. Scientific Data.

For peer review only

2021; **8**. DOI:10.1038/s41597-021-00937-4.

19 Bartl-Pokorny KD, Pokorny FB, Batliner A, *et al.* The voice of COVID-19: Acoustic correlates of infection in sustained vowels. *J Acoust Soc Am* 2021; **149**: 4377.

20 Fagherazzi G, Fischer A, Betsou F, *et al.* Protocol for a prospective, longitudinal cohort of people with COVID-19 and their household members to study factors associated with disease severity: the Predi-COVID study. *BMJ Open* 2020; **10**: e041834.

21 United Nations. Universal Declaration of Human Rights | United Nations. https://www.un.org/en/about-us/universal-declaration-of-human-rights (accessed Nov 18, 2021).

22 Hoffmann W, Latza U, Baumeister SE, *et al.* Guidelines and recommendations for ensuring Good Epidemiological Practice (GEP): a guideline developed by the German Society for Epidemiology. *Eur J Epidemiol* 2019; **34**: 301–17.

23 [datasets] LIHVOICE.Predi_COVID_Fatigue_Vocal_Biomarker/Android_audioset.csv at main · LIHVOICE/Predi_COVID_Fatigue_Vocal_Biomarker. GitHub. https://github.com/LIHVOICE/Predi_COVID_Fatigue_Vocal_Biomarker (accessed Jan 31, 2022).

24 [datasets] Elbéji A, Zhang L, Higa E, *et al.* Audio recordings of COVID-19 positive individuals from the prospective Predi-COVID cohort study with their fatigue status. 2022; published online Feb 1. DOI:10.5281/zenodo.5937844.

25 Barman R, Deshpande S, Agarwal S, Inamdar U, Devare M. Transfer Learning for Small Dataset. 2019; published online March 26. http://dx.doi.org/ (accessed Nov 18, 2021).

26 Weiss K, Khoshgoftaar TM, Wang D. A survey of transfer learning. *Journal of Big Data* 2016; **3**: 1–40.

27 Simonyan K, Zisserman A. Very Deep Convolutional Networks for Large-Scale Image Recognition. 2014; published online Sept 4. http://arxiv.org/abs/1409.1556

For peer review only

(accessed Aug 6, 2021).

28 Hasan BMS, Abdulazeez AM. A Review of Principal Component Analysis Algorithm for Dimensionality Reduction. *Journal of Soft Computing and Data Mining* 2021; **2**: 20–30.

29 König A, Satt A, Sorin A, *et al.* Automatic speech analysis for the assessment of patients with predementia and Alzheimer's disease. *Alzheimer's & dementia (Amsterdam, Netherlands)* 2015; **1**. DOI:10.1016/j.dadm.2014.11.012.

30 Zhang L, Duvvuri R, Chandra KKL, Nguyen T, Ghomi RH. Automated voice biomarkers for depression symptoms using an online cross-sectional data collection initiative. *Depress Anxiety* 2020; **37**. DOI:10.1002/da.23020.

31 Noninvasive Vocal Biomarker is Associated With Severe Acute Respiratory Syndrome Coronavirus 2 Infection. *Mayo Clinic Proceedings: Innovations, Quality & Outcomes* 2021; **5**: 654–62.

32 COVID-19 Sounds App. http://www.covid-19-sounds.org/ (accessed Nov 18, 2021).

33 Goërtz YMJ, Van Herck M, Delbressine JM, *et al.* Persistent symptoms 3 months after a SARS-CoV-2 infection: the post-COVID-19 syndrome? *ERJ Open Res* 2020; **6**. DOI:10.1183/23120541.00542-2020.

34 Townsend L, Dyer AH, Jones K, *et al.* Persistent fatigue following SARS-CoV-2 infection is common and independent of severity of initial infection. *PLoS One* 2020; **15**: e0240784.

35 Arnold P, Njemini R, Vantieghem S, *et al.* Peripheral muscle fatigue in hospitalised geriatric patients is associated with circulating markers of inflammation. Experimental Gerontology. 2017; **95**: 128–35.

36 Fogarty H, Townsend L, Morrin H, *et al.* Persistent endotheliopathy in the pathogenesis of long COVID syndrome. *J Thromb Haemost* 2021; **19**. DOI:10.1111/jth.15490.

37 Pan S-Y, Ding M, Huang J, Cai Y, Huang Y-Z. Airway resistance variation

For peer review only - http://bmjopen.bmj.com/site/about/guidelines.xhtml

correlates with prognosis of critically ill COVID-19 patients: A computational fluid dynamics study. *Comput Methods Programs Biomed* 2021; **208**: 106257.

38 Murry T. The voice and its disorders, 4th edition. By Margaret C. L. Greene, 446 pp, illus, J. B. Lippincott Co., Philadelphia, PA, 1980. $47.50. 1981. DOI:10.1002/HED.2890030517.

39 Zörner S, Kaltenbacher M, Döllinger M. Investigation of prescribed movement in fluid–structure interaction simulation for the human phonation process. Computers & Fluids. 2013; **86**: 133–40.

40 Yin J, Zhang Z. Laryngeal muscular control of vocal fold posturing: Numerical modeling and experimental validation. *J Acoust Soc Am* 2016; **140**: EL280.

41 Dassie-Leite AP, Gueths TP, Ribeiro VV, Pereira EC, Martins P do N, Daniel CR. Vocal Signs and Symptoms Related to COVID-19 and Risk Factors for their Persistence. *J Voice* 2021; published online Aug 11. DOI:10.1016/j.jvoice.2021.07.013.

Legends

Table 1: Study population characteristics
Table 2: Results of the prediction models
Figure 1. General Pipeline
Figure 2. VGG19 Feature Extraction
Figure 3. Derivation of the digital fatigue vocal biomarker for Android and iOS users.
SOM 1. Text to read
SOM 2. VGG19 extracted features from participants' audio recordings

For peer review only

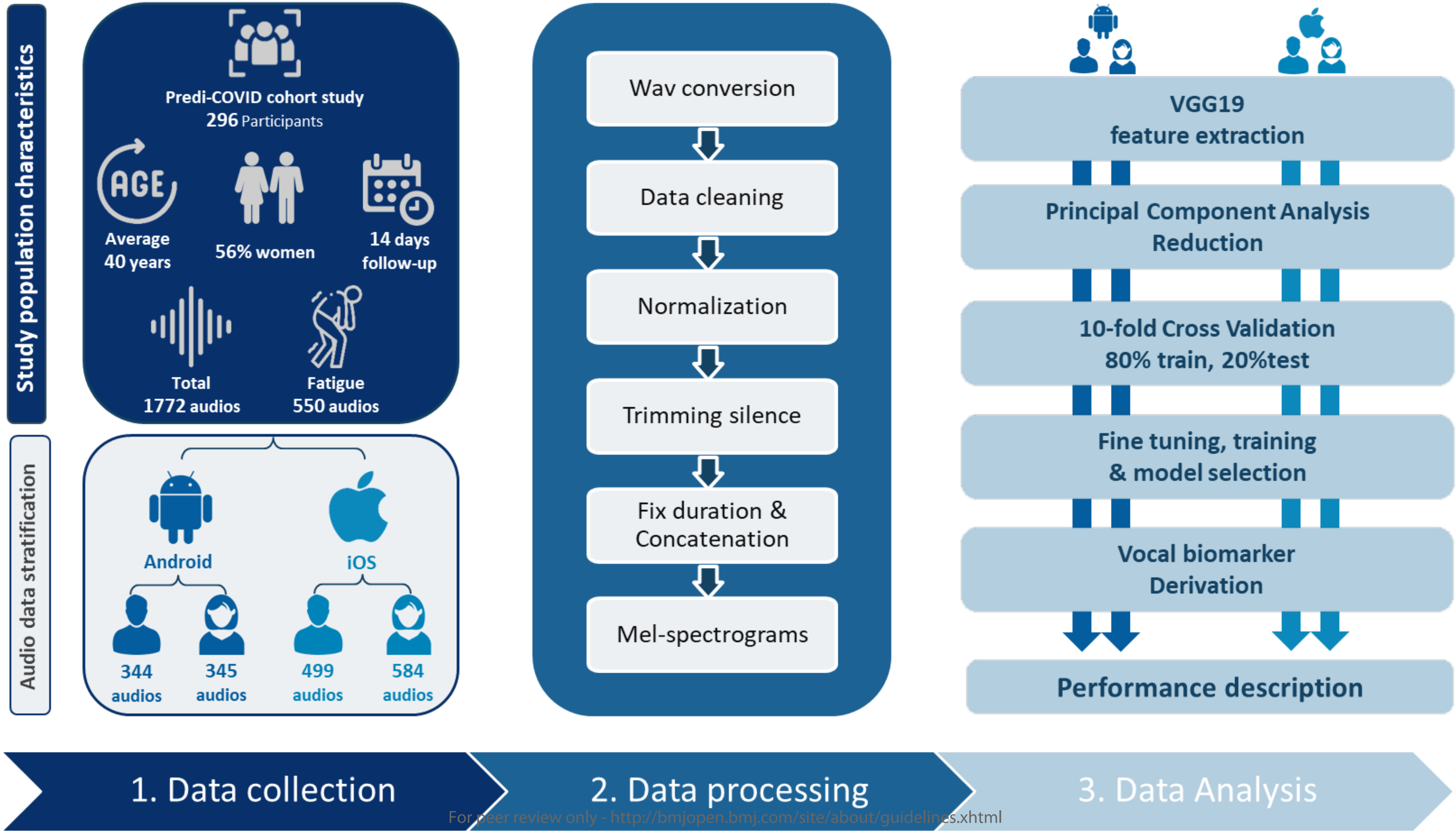
Study population characteristics
Predi-COVID cohort study
296 Participants
AGE
Average
40 years
56% women
14 days
follow-up
Total
1772 audios
Fatigue
550 audios
Audio data stratification
Android
iOS
344
audios
345
audios
499
audios
584
audios
Wav conversion
Data cleaning
Normalization
Trimming silence
Fix duration &
Concatenation
Mel-spectrograms
VGG19
feature extraction
Principal Component Analysis
Reduction
10-fold Cross Validation
80% train, 20%test
Fine tuning, training
& model selection
Vocal biomarker
Derivation
Performance description
1. Data collection
2. Data processing
3. Data Analysis

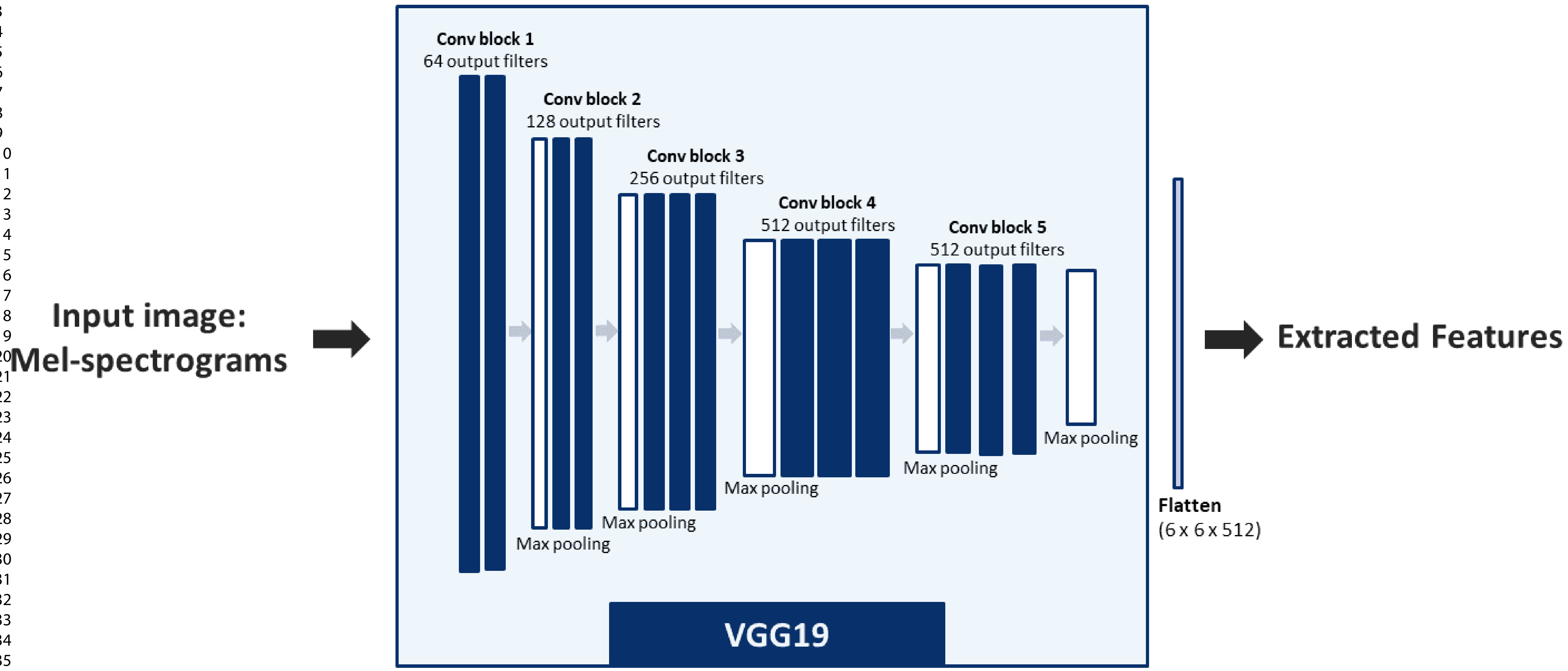
Input image:
Mel-spectrograms
Conv block 1
64 output filters
Conv block 2
128 output filters
Conv block 3
256 output filters
Conv block 4
512 output filters
Conv block 5
512 output filters
Max pooling
Max pooling
Max pooling
Max pooling
Max pooling
VGG19
Flatten
(6 x 6 x 512)
Extracted Features

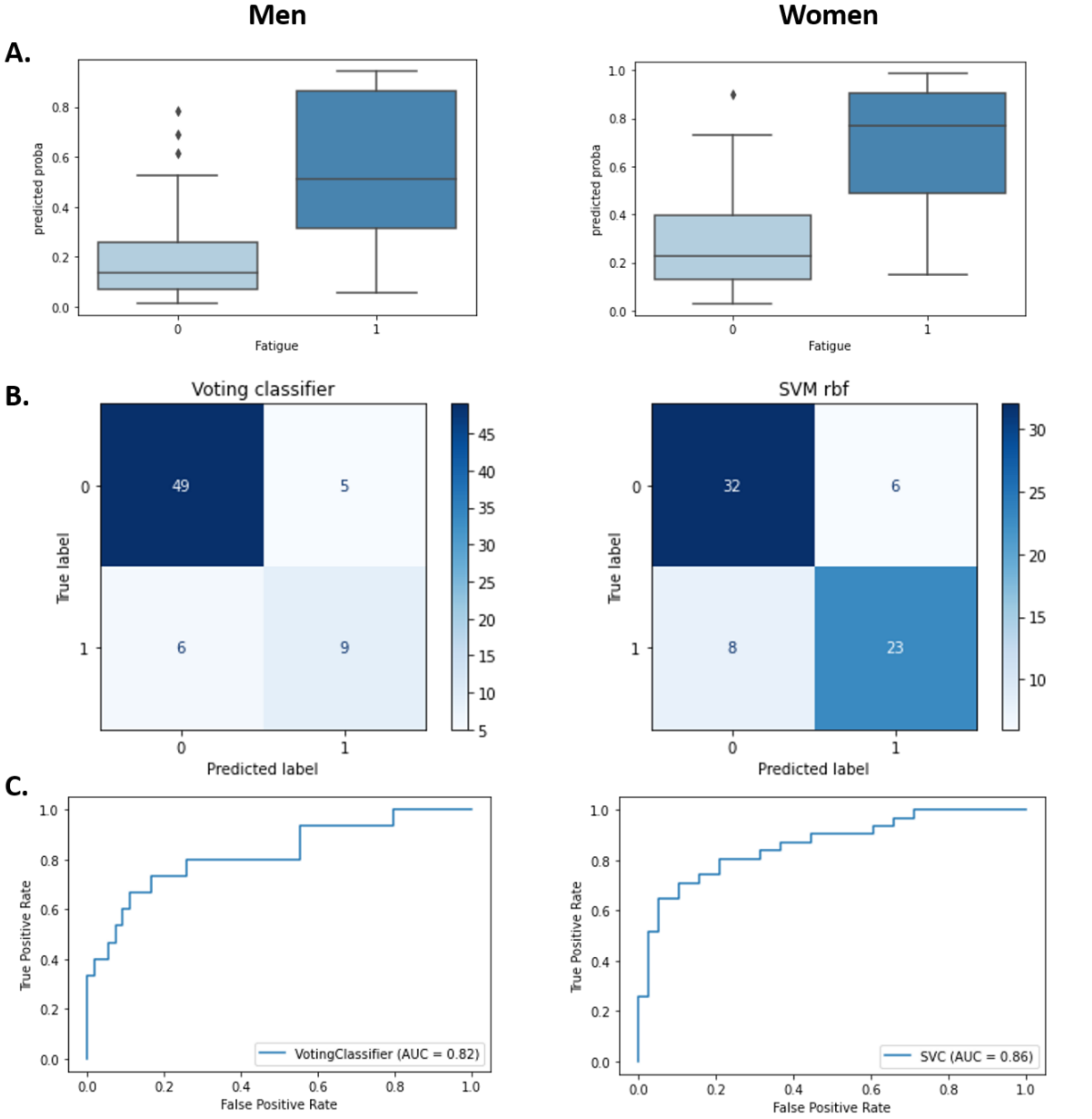

Figure 3a. Derivation of the digital fatigue vocal biomarker for Android users

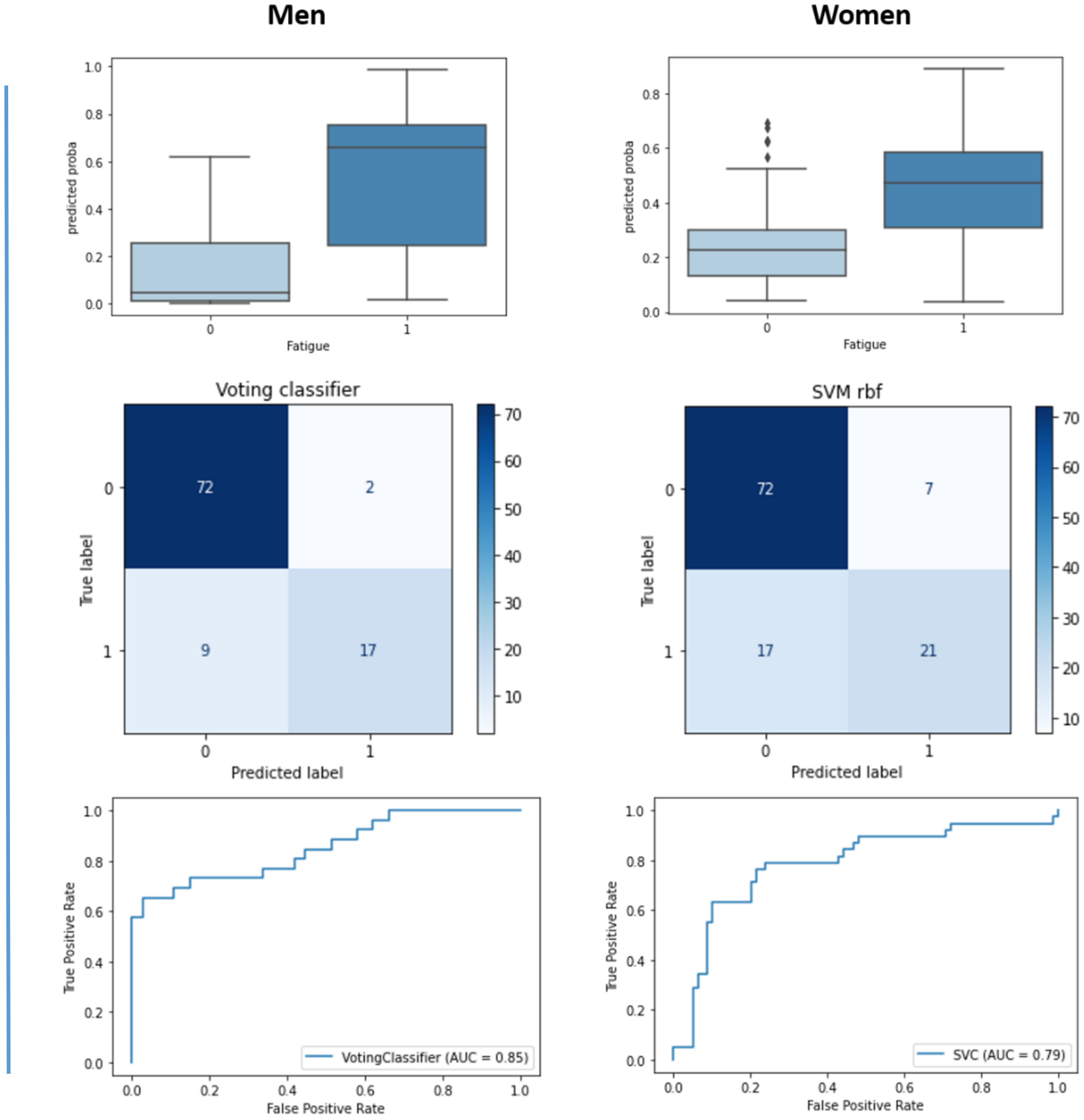

Figure 3b. Derivation of the digital fatigue vocal biomarker for iOS users

Supplementary Online Material 1. Standardized, prespecified text to be read by study participants to collect voice recordings.

Universal Declaration of Human Rights, United Nations.

English

Everyone has the right to a standard of living adequate for the health and well-being of himself and of his family, including food, clothing, housing and medical care and necessary social services, and the right to security in the event of unemployment, sickness, disability, widowhood, old age or other lack of livelihood in circumstances beyond his control.

French

Toute personne a droit à un niveau de vie suffisant pour assurer sa santé, son bien-être et ceux de sa famille, notamment pour l'alimentation, l'habillement, le logement, les soins médicaux ainsi que pour les services sociaux nécessaires ; elle a droit à la sécurité en cas de chômage, de maladie, d'invalidité, de veuvage, de vieillesse ou dans les autres cas de perte de ses moyens de subsistance par suite de circonstances indépendantes de sa volonté.

German

Jeder hat das Recht auf einen Lebensstandard, der seine und seiner Familie Gesundheit und Wohl gewährleistet, einschließlich Nahrung, Kleidung, Wohnung,

ärztliche Versorgung und notwendige soziale Leistungen gewährleistet sowie das Recht auf Sicherheit im Falle von Arbeitslosigkeit, Krankheit, Invalidität oder Verwitwung, im Alter sowie bei anderweitigem Verlust seiner Unterhaltsmittel durch unverschuldete Umstände.

Portuguese

Toda a pessoa tem direito a um nível de vida suficiente para lhe assegurar e à sua família a saúde e o bem-estar, principalmente quanto à alimentação, ao vestuário, ao alojamento, à assistência médica e ainda quanto aos serviços sociais necessários, e tem direito à segurança no desemprego, na doença, na invalidez, na viuvez, na velhice ou noutros casos de perda de meios de subsistência por circunstâncias independentes da sua vontade.

Supplementary Online Material 2. VGG19 extracted features from participants' audio recordings

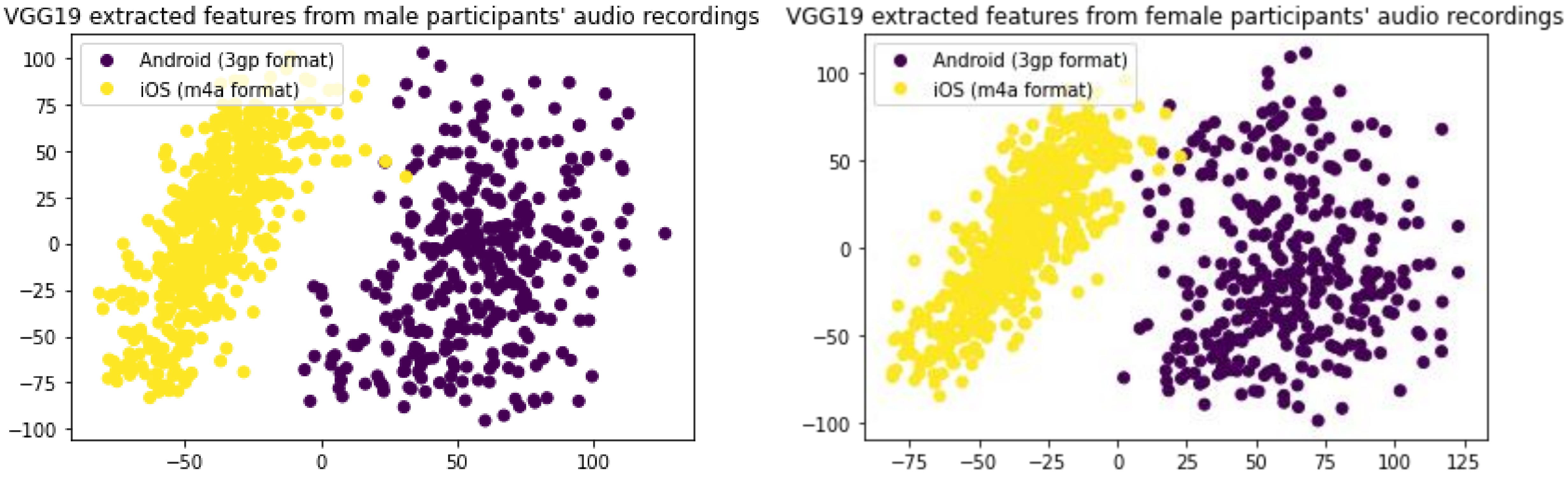

The scatter plot of the 250 relevant components given by PCA reduction revealed two distinct clusters. These two groups appeared to characterize the audio formats, m4a (iOS users) and 3gp (Android users).

It was consequently hypothesized that our data was heterogeneous and that it would be preferable to fit the models with each audio format independently.

For peer review only - http://bmjopen.bmj.com/site/about/guidelines.xhtml

TRIPOD Checklist: Prediction Model Development and Validation

Section/Topic	Item		Checklist Item	Page
**Title and abstract**				
Title	1	D;V	Identify the study as developing and/or validating a multivariable prediction model, the target population, and the outcome to be predicted.	1
Abstract	2	D;V	Provide a summary of objectives, study design, setting, participants, sample size, predictors, outcome, statistical analysis, results, and conclusions.	2
**Introduction**				
Background and objectives	3a	D;V	Explain the medical context (including whether diagnostic or prognostic) and rationale for developing or validating the multivariable prediction model, including references to existing models.	4
	3b	D;V	Specify the objectives, including whether the study describes the development or validation of the model or both.	5
**Methods**				
Source of data	4a	D;V	Describe the study design or source of data (e.g., randomized trial, cohort, or registry data), separately for the development and validation data sets, if applicable.	5
	4b	D;V	Specify the key study dates, including start of accrual; end of accrual; and, if applicable, end of follow-up.	
Participants	5a	D;V	Specify key elements of the study setting (e.g., primary care, secondary care, general population) including number and location of centres.	6
	5b	D;V	Describe eligibility criteria for participants.	
	5c	D;V	Give details of treatments received, if relevant.	
Outcome	6a	D;V	Clearly define the outcome that is predicted by the prediction model, including how and when assessed.	9
	6b	D;V	Report any actions to blind assessment of the outcome to be predicted.	
Predictors	7a	D;V	Clearly define all predictors used in developing or validating the multivariable prediction model, including how and when they were measured.	8
	7b	D;V	Report any actions to blind assessment of predictors for the outcome and other predictors.	
Sample size	8	D;V	Explain how the study size was arrived at.	7
Missing data	9	D;V	Describe how missing data were handled (e.g., complete-case analysis, single imputation, multiple imputation) with details of any imputation method.	
Statistical analysis methods	10a	D	Describe how predictors were handled in the analyses.	8
	10b	D	Specify type of model, all model-building procedures (including any predictor selection), and method for internal validation.	
	10c	V	For validation, describe how the predictions were calculated.	
	10d	D;V	Specify all measures used to assess model performance and, if relevant, to compare multiple models.	
	10e	V	Describe any model updating (e.g., recalibration) arising from the validation, if done.	
Risk groups	11	D;V	Provide details on how risk groups were created, if done.	
Development vs. validation	12	V	For validation, identify any differences from the development data in setting, eligibility criteria, outcome, and predictors.	9
**Results**				
Participants	13a	D;V	Describe the flow of participants through the study, including the number of participants with and without the outcome and, if applicable, a summary of the follow-up time. A diagram may be helpful.	9
	13b	D;V	Describe the characteristics of the participants (basic demographics, clinical features, available predictors), including the number of participants with missing data for predictors and outcome.	
	13c	V	For validation, show a comparison with the development data of the distribution of important variables (demographics, predictors and outcome).	
Model development	14a	D	Specify the number of participants and outcome events in each analysis.	9
	14b	D	If done, report the unadjusted association between each candidate predictor and outcome.	
Model specification	15a	D	Present the full prediction model to allow predictions for individuals (i.e., all regression coefficients, and model intercept or baseline survival at a given time point).	10
	15b	D	Explain how to the use the prediction model.	
Model performance	16	D;V	Report performance measures for the prediction model.	10
Model-updating	17	V	If done, report the results from any model updating (i.e., model specification, model performance).	
**Discussion**				
Limitations	18	D;V	Discuss any limitations of the study (such as nonrepresentative sample, few events per predictor, missing data).	11
Interpretation	19a	V	For validation, discuss the results with reference to performance in the development data, and any other validation data.	10
	19b	D;V	Give an overall interpretation of the results, considering objectives, limitations, results from similar studies, and other relevant evidence.	
Implications	20	D;V	Discuss the potential clinical use of the model and implications for future research.	12
**Other information**				
Supplementary information	21	D;V	Provide information about the availability of supplementary resources, such as study protocol, Web calculator, and data sets.	5,6,10
Funding	22	D;V	Give the source of funding and the role of the funders for the present study.	3

*Items relevant only to the development of a prediction model are denoted by D, items relating solely to a validation of a prediction model are denoted by V, and items relating to both are denoted D;V. We recommend using the TRIPOD Checklist in conjunction with the TRIPOD Explanation and Elaboration document.

For peer review only - http://bmjopen.bmj.com/site/about/guidelines.xhtml

Vocal biomarker predicts fatigue in people with COVID-19: results from the prospective Predi-COVID cohort study

Journal:	*BMJ Open*
Manuscript ID	bmjopen-2022-062463.R2
Article Type:	Original research
Date Submitted by the Author:	20-Oct-2022

Complete List of Authors:	Elbéji, Abir; Luxembourg Institute of Health, Department of Precision Health<br>Zhang, Lu; Luxembourg Institute of Health, Department of Precision Health<br>Higa, Eduardo; Luxembourg Institute of Health, Department of Precision Health<br>Fischer, Aurélie; Luxembourg Institute of Health, Departement of Population Health<br>Despotovic, Vladimir; Luxembourg Institute of Health, Department of Precision Health<br>Nazarov, Petr V.; Luxembourg Institute of Health, Department of Precision Health<br>Aguayo, Gloria; Luxembourg Institute of Health, Department of Population Health<br>Fagherazzi, Guy; Luxembourg Institute of Health, Department of Precision Health

**Primary Subject Heading**:	Health informatics
Secondary Subject Heading:	Global health
Keywords:	COVID-19, Health informatics < BIOTECHNOLOGY & BIOINFORMATICS, Public health < INFECTIOUS DISEASES

SCHOLARONE™ Manuscripts

I, the Submitting Author has the right to grant and does grant on behalf of all authors of the Work (as defined in the below author licence), an exclusive licence and/or a non-exclusive licence for contributions from authors who are: i) UK Crown employees; ii) where BMJ has agreed a CC-BY licence shall apply, and/or iii) in accordance with the terms applicable for US Federal Government officers or employees acting as part of their official duties; on a worldwide, perpetual, irrevocable, royalty-free basis to BMJ Publishing Group Ltd ("BMJ") its licensees and where the relevant Journal is co-owned by BMJ to the co-owners of the Journal, to publish the Work in this journal and any other BMJ products and to exploit all rights, as set out in our licence.

The Submitting Author accepts and understands that any supply made under these terms is made by BMJ to the Submitting Author unless you are acting as an employee on behalf of your employer or a postgraduate student of an affiliated institution which is paying any applicable article publishing charge ("APC") for Open Access articles. Where the Submitting Author wishes to make the Work available on an Open Access basis (and intends to pay the relevant APC), the terms of reuse of such Open Access shall be governed by a Creative Commons licence – details of these licences and which Creative Commons licence will apply to this Work are set out in our licence referred to above.

Other than as permitted in any relevant BMJ Author's Self Archiving Policies, I confirm this Work has not been accepted for publication elsewhere, is not being considered for publication elsewhere and does not duplicate material already published. I confirm all authors consent to publication of this Work and authorise the granting of this licence.

For peer review only

Vocal biomarker predicts fatigue in people with COVID-19: results from the prospective Predi-COVID cohort study

Abir Elbéji[1], Lu Zhang[2], Eduardo Higa[1], Aurélie Fischer[1], Vladimir Despotovic[2], Petr V. Nazarov[2], Gloria A. Aguayo[1], Guy Fagherazzi[1]

1. Deep Digital Phenotyping Research Unit. Department of Precision Health. Luxembourg Institute of Health, 1 A-B rue Thomas Edison, L-1445 Strassen, Luxembourg
2. Bioinformatics Platform. Luxembourg Institute of Health, 1A-B, rue Thomas Edison, L-1445 Strassen, Luxembourg

Reprints and correspondence:

Dr Guy Fagherazzi, PhD, ADR
Director of Department of Precision Health
Head of the Deep Digital Phenotyping Research Unit
Luxembourg Institute of Health
1 AB rue Thomas Edison, L-1445 STRASSEN, LUXEMBOURG
Phone: +352 6 21 58 95 54
Email: guy.fagherazzi@lih.lu
Website: https://ddp.lih.lu/

For peer review only

Abstract

Objective

To develop a vocal biomarker for fatigue monitoring in people with COVID-19.

Design Prospective cohort study.

Setting Predi-COVID data between May 2020 and May 2021.

Participants

A total of 1772 voice recordings was used to train an AI-based algorithm to predict fatigue, stratified by gender and smartphone's operating system (Android/iOS). The recordings were collected from 296 participants tracked for two weeks following SARS-CoV-2 infection.

primary and secondary outcome measures

Four machine learning algorithms (Logistic regression, k-nearest neighbors, support vector machine, and soft voting classifier) were used to train and derive the fatigue vocal biomarker. The models were evaluated based on the following metrics: Area Under the ROC curve (AUC), accuracy, F1-score, precision, and recall. The Brier score was also used to evaluate the models' calibrations.

Results

The final study population included 56% of women and had a mean (±SD) age of 40 (±13) years. Women were more likely to report fatigue (*P<.001*). We developed four models for Android female, Android male, iOS female, and iOS male users with a weighted AUC of 86%, 82%, 79%, 85%, and a mean Brier Score of 0.15, 0.12, 0.17, 0.12, respectively. The vocal biomarker derived from the prediction models successfully discriminated COVID-19 participants with and without fatigue.

Conclusions

This study demonstrates the feasibility of identifying and remotely monitoring fatigue thanks to voice. Vocal biomarkers, digitally integrated into telemedicine technologies, are expected to improve the monitoring of people with COVID-19 or Long-COVID.

ClinicalTrials.gov Identifier: NCT04380987

For peer review only - http://bmjopen.bmj.com/site/about/guidelines.xhtml

Strengths and limitations

-This is the first study supporting the hypothesis that fatigue can be accurately monitored based on voice in people with COVID-19.

-The analyses were based on a multilingual database of standardized voice recordings collected in real-life from people with confirmed SARS-CoV-2 infection as determined by PCR.

-There is no similar dataset available yet in the literature to replicate our findings.

-The vocal biomarker is trained on a binary outcome (Fatigue, Yes/No) and does not reflect the entire spectrum of fatigue severity. Further work should be performed in that direction.

For peer review only

Introduction

Coronavirus disease 2019 (COVID-19) is a global outbreak. More than 199 million confirmed cases of COVID-19 have been detected worldwide as of 4 August 2021, with more than 4 million deaths reported by the World Health Organization[1]. The worldwide population and healthcare systems have been greatly impacted by the COVID-19 pandemic. The pandemic has essentially put whole healthcare systems under pressure, requiring national or regional lockdowns[2]. Finding solutions that allow healthcare providers to focus on the more important and urgent patients, was, and still is, critical.

This outbreak continues to impact people, with many patients suffering from a range of acute symptoms, such as fatigue. Fatigue is a common symptom in patients with COVID-19 that can impact their quality of life, treatment adherence, and can be associated with numerous complications[3]. Recent findings showed that fatigue is a major symptom of the frequently reported Long-COVID syndrome. After recovering from the acute disease caused by the SARS outbreak, up to 60% of patients reported chronic fatigue 12 months later[4]. This supports the need for long-term monitoring solutions for these patients.

In general, fatigue can be of two types: physical and mental[5] experiencing lack of energy, inability to start and perform everyday activities, and lack of desire to do things. In the context of COVID-19, determinants of fatigue were categorized as both central and psychological factors, the latest might also be indirectly caused by pandemic-related fear and anxiety[6,7].

Fatigue affects men and women differently and has previously been shown to be reported differently in the two genders. Men and women have different anatomy and physiology, resulting in significant sex differences in fatigability[8].

Telemedicine, artificial intelligence (AI), and big data predictive analytics are examples of digital health technologies that have the potential to minimize the damaging effects of COVID-19 by improving responses to public health problems at a population level[9]. Using telemonitoring technologies to enable self-surveillance and remote monitoring of symptoms might therefore help to improve and personalize COVID-19 care delivery[10].

For peer review only

Voice is a promising source of digital data since it is rich, user-friendly, inexpensive to collect, and non-invasive, and can be used to develop vocal biomarkers that characterize disease states. Previous research was mostly conducted in the field of neurodegenerative diseases, such as Parkinson's disease[11] and Alzheimer's disease[12]. There are also studies that confirm the relation of voice disorders to fatigue, e.g., in Chronic Fatigue Syndrome (CFS). Neuromuscular, neuropsychological and hormonal dysfunction associated with CFS can influence the phonation and articulation, and alter tension, viscosity and thickness of the tissue of the larynx, tongue and lips, leading to decreased voice quality[13]. Increased fatigue affects voice characteristics, such as pitch, word duration[14] and timing of articulated sounds[15]. Vocal changes related to fatigue are more observed in consonant sounds that require a high average airflow[16].

In the context of the COVID-19 pandemic, respiratory sounds (e.g coughs, breathing, and voice) are also used as sources of information to develop COVID-19 screening tools[17,18,19]. However, no previous work has been devoted to investigating the association of voice with COVID-19 symptoms.

We hypothesized that there is an association between fatigue and voice in patients with COVID-19 and that it is possible to train an AI-based model to identify fatigue and subsequently generate a digital vocal biomarker for fatigue monitoring. We used data from the large hybrid prospective Predi-COVID cohort study to investigate this hypothesis.

Methods

Study design

This project uses data from the Predi-COVID study[20]. Predi-COVID is a hybrid cohort study that started in May 2020 in Luxembourg and involved participants who should meet all of the following requirements: (1) a signed informed consent form; (2) participants with confirmed SARS-CoV-2 infection as determined by PCR at one of Luxembourg's certified laboratories; and (3) 18 years and older.

This study combines data from the national surveillance system, which is used for virtually all COVID-19 positive patients. Biological sampling, electronic patient-

For peer review only - http://bmjopen.bmj.com/site/about/guidelines.xhtml

reported outcomes, and smartphone voice recording were collected to identify vocal biomarkers of respiratory syndromes and fatigue in this study. More details about the Predi-COVID study can be found elsewhere[20].

Health Inspection collaborators made the initial phone contact with potential participants. Those who consented to participate were contacted by a qualified nurse from the Clinical and Epidemiological Investigation Center (CIEC - Luxembourg Institute of Health), who outlined the study and arranged home or hospital visits.

Patient and Public Involvement

The Predi-COVID initiative was an emergency response from national research institutions grouped under 'Research Luxembourg' to fight the COVID-19 pandemic in Luxembourg and contribute to the general effort in the crisis. Therefore, for timing and safety reasons, patients with COVID-19 were not directly included to participate in the study design. However, the first participants included in Predi-COVID provided feedback on general workflow, data collection, questionnaires, and sampling, which was taken into account in an amendment to the protocol[20].

Data collection

Participants were followed for up to a year using a smartphone app to collect voice data. To ensure a minimum quality level, participants were asked to record it in a quiet environment while maintaining a certain distance from the microphone, and an audio example of what was required was also provided.

All the participants of this study were invited to record two audio types. The first, Type 1 audio, required participants to read paragraph 1 of article 25 of the Declaration of Human Rights[21], in their preferred language: French, German, English, or Portuguese; and the second, Type 2 audio, required them to hold the [a] vowel phonation without breathing for as long as they could (see Supplementary Online Material 1 for more details).

Predi-COVID collects data in conformity with the German Society of Epidemiology's best practices guidelines[22]. To draft the manuscript, we followed the TRIPOD criteria

For peer review only

for reporting AI-based model development and validation, as well as the corresponding checklist.

All Predi-COVID participants recruited between May 2020 and May 2021 who reported their fatigue status ("I feel well" as "No Fatigue" and "I am fatigued"/"I don't feel well" as "Fatigue") on the same day as the audio recordings during the 14 days of follow-up were included in this study[23]. As a result, several audio recordings for a single participant were available for both audio types[24].

Audio characteristics and vocal biomarker training

The audio recordings were collected in two formats, 3gp format (Android devices) and m4a format (iOS devices). Based on the smartphone's operating system and the user's gender (male/female), we trained one model for each category. This stratification was performed to minimize data heterogeneity and deal with sex as a potential confounding bias.

Audio pre-processing

All of the raw audio recordings were pre-processed (Figure 1). They were initially converted to .wav files, with audios lasting less than 2 seconds being excluded. Then, an audio clustering (DBSCAN) on basic features (duration, average, sum, and standard deviation of signal power, and fundamental frequency) was performed to detect outliers that were manually checked while excluding poor quality audios with 1) too noisy, 2) incorrect text reading, 3) type 1 and type 2 audios mixed, or 4) extended silence in the middle**.** Finally, peak normalization was used to boost the volume of quiet audio segments, and leading and trailing silences longer than 350 milliseconds were trimmed.

Feature extraction

We used transfer learning for the feature extraction process since it is adapted for small training databases[25]. Transfer learning is a technique where a model is constructed and trained with a set containing a large amount of data and then transfer and apply this learning to our dataset on top of it. It has the advantage of reducing the amount of data required while shortening training time and improving performance when compared to models built from scratch[26].

For peer review only - http://bmjopen.bmj.com/site/about/guidelines.xhtml

Convolutional neural networks require a fixed input size, whereas audio instances in our dataset were of variable length. To deal with this issue, Zero-padding was used to set the duration of each audio file to 50 seconds (the maximum length in our database). To raise the amount of information fed to the classifiers, type 1 (text reading) and type 2 ([a] phonation) audios were concatenated and used as a single input to the learning models.

All the audio recordings were first resampled to 8kHz and then converted to Mel-spectrograms using the Librosa library in Python. The hop-length was 2048 samples, and the number of Mel coefficients was set to 196. The Mel spectrograms were passed through VGG19 convolutional neural network architecture provided by Keras, which was pre-trained on the ImageNet database[27]. This approach, presented in Figure 2, may be considered as a feature extraction step, as it converts audio recordings to 512 feature maps, each of a size 6x6, leading to a total of 18432 features.

This large number of features is computationally expensive. Principal Component Analysis (PCA)[28] is therefore used for dimensionality reduction and to select the number of relevant components explaining the maximum of the variance in the data.

For peer review only

Statistical analysis

We divided our data into “Fatigue” and “No Fatigue” groups based on the participant’s reported answers for the inclusion and daily fatigue assessment of Predi-COVID. To characterize participants, descriptive statistics were used, which included means, standard deviations for quantitative variables, and counts and percentages for qualitative variables. The two population groups (3gp (Android users) and m4a (iOS users)) were compared using a student test for continuous variables, and a χ2 test for categorical variables.

A 10-fold cross-validation procedure was conducted on the training cohort participants to evaluate four classification models (logistic regression (LR), k-nearest neighbors (KNN), support vector machine (SVM), and soft voting classifier (VC), scikit-learn implementation in Python) at different regularization levels via a grid search, with the following evaluation metrics: area under the ROC curve (AUC), accuracy, F1-score, precision, and recall. The Brier score was also used to evaluate the calibration of the selected models.

The predicted probability of being classified as fatigued from the best model was considered as our final vocal biomarker, which may be used as a quantitative metric to monitor fatigue.

Results

Study population characteristics

The final study population is composed of 296 participants of whom 165 were women (56%), with an average age of 40 years (SD = 13). To record both audio types,109 (37%) participants utilized Android smartphones (3gp format), whereas 187 (63%) used iOS devices (m4a format). We found no difference in the distribution of age, gender, body mass index, smoking, antibiotic usage, and asthma, between the two types of devices (*P-value>.05*). The overall rate of comorbidities in this study was relatively low: there were 31 (10%) participants who used antibiotics and only 12 (4%) participants with asthma. More details are shown in Table 1.

Table 1: Study population characteristics

For peer review only - http://bmjopen.bmj.com/site/about/guidelines.xhtml

The clinical data in the table above describe the overall population of the study. The total number and its percentage are used to represent all categorical data. The table below summarizes general information for describing audio data.

All p-values comparing iOS (m4a) and Android users (3gp) were calculated using chi2 and Student's t-tests.

		**All**	**m4a**		**3gp**		**P-values (m4a, 3gp)**
			**Female**	**Male**	**Female**	**Male**	
**Participants (N)**	Total	296	107	80	51	58	-
**Age (years)**	mean (SD)	40.3 (12.6)	38.8 (13.4)	42.9 (12.7)	37.8 (11.6)	41.5 (11.3)	0.28
**Body Mass Index (kg/m²)**	mean (SD)	24.1 (4.7)	24.6 (5.5)	26.5 (4.1)	24.1 (3.8)	26.6 (4.17)	0.95
**Antibiotic (%)**	No	265 (90%)	93 (87%)	73 (91%)	44 (86%)	55 (95%)	0.87
	Yes	31 (10%)	14 (13%)	7 (9%)	7 (14%)	3 (5%)	
**Asthma (%)**	No	284 (96%)	104 (97%)	75 (94%)	47 (92%)	58 (100%)	0.82
	Yes	12 (4%)	3 (3%)	5 (6%)	4 (8%)	0 (0%)	
**Smoking (%)**	Never	199 (67%)	77 (72%)	51 (64%)	36 (71%)	35 (60%)	0.41
	Former smoker	53 (18%)	19 (18%)	20 (25%)	9 (18%)	13 (22%)	
	Current smoker	44 (15%)	11 (10%)	9 (11%)	6 (11%)	10 (18%)	
**Audio recordings**	Total	1772	584	499	345	344	<0.001
	No Fatigue	1222 (69%)	394 (67%)	370 (74%)	190 (55%)	268 (78%)	
	Fatigue	550 (31%)	190 (33%)	129 (26%)	155 (45%)	76 (22%)	
**Mean (SD) and maximum of audio recording per participant in the 14-day follow-up period**	mean (SD)	6 (5)	6 (5)	6 (5)	6 (5)	6 (5)	-
	max	16	14	16	15	14	

Participants reported their fatigue status on average 6 days during the first 14 days of follow-up, resulting in the analysis of 1772 audio recordings for each audio type (type 1 and type 2) when all inclusion criteria were met, including 550 audio recordings for

For peer review only - http://bmjopen.bmj.com/site/about/guidelines.xhtml

participants with fatigue. In both audio sets, women reported experiencing fatigue at a higher rate than men (*P-value<.001*). Women constituted 155 (60%) of all fatigued Android users and 190 (67%) of all fatigued iOS users.

Prediction models

We reduced the extracted features from Mel-spectrograms to 250 top components with PCA, explaining 97% and 99% of the variance in the data for iOS and Android audio sets respectively. We then compared the performances of the machine learning algorithms to select the best models for the derivation of the vocal biomarkers.

The voting classifier was the best model selected for the development of the vocal biomarker for male iOS users, with an AUC of 85% and overall accuracy, precision, recall, and f1-score of 89%. The model selected for female iOS users was SVM with an overall precision of 79% and an AUC of 79%. For male Android users, the selected model is the voting classifier with precision, recall an f1-score of 84%, and a weighted AUC of 82%. For female Android users, the SVM was selected with an overall precision of 80% and an AUC of 86%. More details are shown in Table 2.

As shown in Figure 3, the calibrations of the selected models were good (Mean Brier Scores = 0.15, 0.12, 0.17, and 0.12 respectively for Android female users, Android male users, iOS female users, and iOS male users).

Derivation of the digital fatigue vocal biomarker

Based on the model selected for each audio set, we derived the trained vocal biomarkers which quantitatively represent the probability of being labeled as fatigued.

For peer review only - http://bmjopen.bmj.com/site/about/guidelines.xhtml

Table 2: Results of the prediction models

The selected models were selected using Recall_1 and weighted AUC and are highlighted in bold. Class 0: No fatigue, Class 1: Fatigue

Audio_format	Gender	ML model	Accuracy	Ov.Precision	Precision_0	Precision_1	Ov.Recall	Recall_0	Recall_1	Ov.f1score	f1-score_0	f1-score_1	Weighted AUC
3gp (Android)	Female	LR	0.77	0.77	0.81	0.73	0.77	0.76	0.77	0.77	0.78	0.75	0.85
		KNN	0.72	0.73	0.70	0.77	0.72	0.87	0.55	0.72	0.78	0.64	0.76
		**SVM**	**0.80**	**0.80**	**0.80**	**0.79**	**0.80**	**0.84**	**0.74**	**0.80**	**0.82**	**0.77**	**0.86**
		VC	0.78	0.78	0.81	0.75	0.78	0.79	0.77	0.78	0.80	0.76	0.86
	Male	LR	0.78	0.79	0.87	0.50	0.78	0.85	0.53	0.79	0.86	0.52	0.81
		KNN	0.83	0.83	0.83	0.80	0.83	0.98	0.27	0.79	0.90	0.40	0.84
		SVM	0.84	0.83	0.88	0.67	0.84	0.93	0.53	0.83	0.90	0.59	0.82
		**VC**	**0.84**	**0.84**	**0.89**	**0.64**	**0.84**	**0.91**	**0.60**	**0.84**	**0.90**	**0.62**	**0.82**
m4a (iOS)	Female	LR	0.72	0.72	0.80	0.56	0.72	0.77	0.61	0.72	0.79	0.58	0.75
		KNN	0.68	0.65	0.72	0.50	0.68	0.86	0.29	0.65	0.78	0.37	0.67
		**SVM**	**0.79**	**0.79**	**0.81**	**0.75**	**0.79**	**0.91**	**0.55**	**0.79**	**0.86**	**0.64**	**0.79**
		VC	0.77	0.76	0.80	0.69	0.77	0.89	0.53	0.76	0.84	0.60	0.78
	Male	LR	0.73	0.74	0.83	0.48	0.73	0.80	0.54	0.73	0.81	0.51	0.80
		KNN	0.89	0.89	0.89	0.89	0.89	0.97	0.65	0.88	0.93	0.76	0.81
		SVM	0.85	0.84	0.86	0.76	0.85	0.95	0.58	0.84	0.90	0.67	0.85
		**VC**	**0.89**	**0.89**	**0.89**	**0.89**	**0.89**	**0.97**	**0.65**	**0.88**	**0.93**	**0.76**	**0.85**

KNN: K-Nearest Neighbors, LR: Logistic Regression, Ov. : Overall, SVM: Support Vector Machine, VC: Voting Classifier

For peer review only

Discussion

In this study, we built an AI-based pipeline to develop a vocal biomarker for both genders and both types of smartphones (male/female, Android/iOS) that effectively recognize fatigued and non-fatigued participants with COVID-19.

We stratified the data to prevent data heterogeneity, which is considered contamination and makes it difficult to build a reliable and consistent classification model(s), resulting in poorer prediction performance. This contamination is caused by two factors: first, significant gender differences in fatigability, since it has previously been shown that men and women experience and report fatigue differently, and second, different microphone types incorporated in both smartphone devices used by the participants (iOS and Android), which have a direct impact on the quality of the recorded audios (machine learning algorithms separate the audio formats rather than the fatigue status if there is no constant microphone. (see Supplementary Online Material 2 for more details).

With the increased interest in remote voice analysis as a noninvasive and powerful telemedicine tool, various studies have been carried out, mostly in neurological disorders (eg, Parkinson's disease[11] and Alzheimer's disease[29]) and mental health (eg. stress and depression[30]). Recently, a significant research effort has evolved to employ respiratory sounds for COVID-19 and the main focus was on the use of cough[17,31] and breathing[32] to develop a COVID-19 screening tool. However, no previous work has been devoted to investigating the association of voice with COVID-19 symptoms, precisely fatigue.

Fatigue is one of the commonly reported symptoms of COVID-19 and Long-COVID syndrome[33], which can persist regardless of how severe COVID-19's acute stage is[34]. A variety of cerebral, peripheral, and psychosocial factors[35,7] play a role in the development of fatigue. It may also occur from chronic inflammation in the brain and at neuromuscular junctions. New evidence shows that patients with Long-COVID syndrome continue to have higher measures of blood clotting, thrombosis[36], which may also explain the persistence of fatigue. COVID-19 is associated with variations in airway resistance[37]. This narrowing of the airway is manifested in the increase in

For peer review only - http://bmjopen.bmj.com/site/about/guidelines.xhtml

audible turbulence in both sighing and yawning, which is frequently associated with fatigue[38].

Human voice is produced by the flow of air from the lungs through the larynx, which causes the vocal fold vibrations, generating a pulsating airstream[39]. The process is controlled by the laryngeal muscle activation[40] but involves the entire respiratory system to provide the air pressure necessary for phonation. Decreased pulmonary function in COVID-19 patients can cause reduced glottal airflow that is essential for normal voice production[41]. Furthermore, in case of increased fatigue, the voice production process may be additionally disturbed due to reduced laryngeal muscle tension, resulting in dysphonia that appears in up to 49% of COVID-19 patients[41].

Study Limitations

This study has several limitations. First, although our data was stratified based on gender and smartphone devices, the mix of languages might also result in different voice features subsequently, in different model performances. There is presently no comparable dataset with similar audio recordings for further external validation of our findings. Thus, more data should be collected to improve the transferability of our vocal biomarker to other populations. Second, our data labeling was only based on a qualitative self-reported fatigue status. A fatigue severity scale would allow a quantitative assessment of fatigue severity in a uniform and unbiased way throughout all participants. Finally, time series voice analysis for each participant was not included in the study. More investigation, including time series analysis, would establish a personalized baseline for each participant, potentially enhancing the performance of our vocal biomarkers.

Conclusion

In this study, we demonstrated the association between fatigue and voice in people with COVID-19 and developed a fatigue vocal biomarker that can accurately predict the presence of fatigue. These findings suggest that vocal biomarkers, digitally incorporated into telemonitoring technologies, might be used to identify and remotely monitor this symptom in patients suffering from COVID-19 as well as other chronic diseases.

For peer review only - http://bmjopen.bmj.com/site/about/guidelines.xhtml

Acknowledgments

We thank all participants that accepted to be involved in the study, members that collaborated to the launch and monitoring of the Predi-COVID cohort, as well as its scientific committee, the IT team responsible for the development of the application, and the nurses in charge of recruitment, data collection, and management on the field.

Contributors

Elbéji and Fagherazzi had full access to all of the data in the study and took responsibility for the integrity of the data and the accuracy of the data analysis.
Fagherazzi, Zhang, and Fischer conceptualized and designed the study.
Elbéji, Zhang, Higa, Fischer, Despotovic, Nazarov, Aguayo, and Fagherazzi collected and analyzed data and contributed to the interpretation.
The statistical analysis was carried out by Elbéji, Zhang, Higa, and Fischer.
Elbéji drafted the initial manuscript. Elbéji, Zhang, Higa, Fischer, Despotovic, Nazarov, Aguayo, and Fagherazzi critically revised the manuscript for more important intellectual content. The funding was obtained by Fagherazzi. Fischer provided administrative, technical, and material support. The corresponding author certifies that all listed authors fulfill the authorship criteria and that no other authors that meet the criteria have been omitted.

Funding Statement

The Predi-COVID study is supported by the Luxembourg National Research Fund (FNR) (Predi-COVID, grant number 14716273), the André Losch Foundation, and the Luxembourg Institute of Health.

Competing interests: None declared.

For peer review only

Data Sharing Statement

Audio data, datasets and source code used in this study are publicly available. Audio data available in Zenodo repository, [DOI: 10.5281/zenodo.5937844] Datasets and source code available in Github, [https://github.com/LIHVOICE/Predi_COVID_Fatigue_Vocal_Biomarker].

Ethics Statement

The National Research Ethics Committee of Luxembourg (study number 202003/07) provided ethics approval to the study in April 2020.

For peer review only

References

1 WHO Coronavirus (COVID-19) Dashboard. https://covid19.who.int (accessed Aug 5, 2021).

2 Website. https://www.oecd.org/coronavirus/policy-responses/the-territorial-impact-of-covid-19-managing-the-crisis-and-recovery-across-levels-of-government-a2c6abaf/.

3 Qi R, Chen W, Liu S, *et al.* Psychological morbidities and fatigue in patients with confirmed COVID-19 during disease outbreak: prevalence and associated biopsychosocial risk factors. *medRxiv* 2020; : 2020.05.08.20031666.

4 Tansey CM, Louie M, Loeb M, *et al.* One-year outcomes and health care utilization in survivors of severe acute respiratory syndrome. *Arch Intern Med* 2007; **167**: 1312–20.

5 Neuropsychological and neurophysiological correlates of fatigue in post-acute patients with neurological manifestations of COVID-19: Insights into a challenging symptom. *J Neurol Sci* 2021; **420**: 117271.

6 Rudroff T, Fietsam AC, Deters JR, Bryant AD, Kamholz J. Post-COVID-19 Fatigue: Potential Contributing Factors. *Brain Sciences* 2020; **10**: 1012.

7 Morgul E, Bener A, Atak M, *et al.* COVID-19 pandemic and psychological fatigue in Turkey. International Journal of Social Psychiatry. 2021; **67**: 128–35.

8 Hunter SK. Sex differences in human fatigability: mechanisms and insight to physiological responses. *Acta Physiol* 2014; **210**: 768–89.

9 Gunasekeran DV, Tseng RMW, Tham Y-C, Wong TY. Applications of digital health for public health responses to COVID-19: a systematic scoping review of artificial intelligence, telehealth and related technologies. *NPJ Digital Medicine* 2021; **4**. DOI:10.1038/s41746-021-00412-9.

10 DeMerle K, Angus DC, Seymour CW. Precision Medicine for COVID-19: Phenotype Anarchy or Promise Realized? *JAMA* 2021; **325**: 2041–2.

11 Tracy JM, Özkanca Y, Atkins DC, Hosseini GR. Investigating voice as a biomarker: Deep phenotyping methods for early detection of Parkinson's disease. *J Biomed Inform* 2020; **104**. DOI:10.1016/j.jbi.2019.103362.

12 Laguarta J, Subirana B. Longitudinal Speech Biomarkers for Automated Alzheimer's Detection. Frontiers in Computer Science. 2021; **3**. DOI:10.3389/fcomp.2021.624694.

13 Cho S-W, Yin CS, Park Y-B, Park Y-J. Differences in self-rated, perceived, and acoustic voice qualities between high- and low-fatigue groups. *J Voice* 2011; **25**: 544–52.

14 Whitmore J, Fisher S. Speech during sustained operations. Speech Communication. 1996; **20**: 55–70.

15 Vollrath M. Automatic measurement of aspects of speech reflecting motor coordination. Behavior Research Methods, Instruments, & Computers. 1994; **26**: 35–40.

16 Greeley HP, Berg J, Friets E, *et al.* Fatigue estimation using voice analysis. Behavior Research Methods. 2007; **39**: 610–9.

17 Detection of COVID-19 from voice, cough and breathing patterns: Dataset and preliminary results. *Comput Biol Med* 2021; **138**: 104944.

18 Orlandic L, Teijeiro T, Atienza D. The COUGHVID crowdsourcing dataset, a corpus for the study of large-scale cough analysis algorithms. Scientific Data.

For peer review only

2021; **8**. DOI:10.1038/s41597-021-00937-4.

19 Bartl-Pokorny KD, Pokorny FB, Batliner A, *et al.* The voice of COVID-19: Acoustic correlates of infection in sustained vowels. *J Acoust Soc Am* 2021; **149**: 4377.

20 Fagherazzi G, Fischer A, Betsou F, *et al.* Protocol for a prospective, longitudinal cohort of people with COVID-19 and their household members to study factors associated with disease severity: the Predi-COVID study. *BMJ Open* 2020; **10**: e041834.

21 United Nations. Universal Declaration of Human Rights | United Nations. https://www.un.org/en/about-us/universal-declaration-of-human-rights (accessed Nov 18, 2021).

22 Hoffmann W, Latza U, Baumeister SE, *et al.* Guidelines and recommendations for ensuring Good Epidemiological Practice (GEP): a guideline developed by the German Society for Epidemiology. *Eur J Epidemiol* 2019; **34**: 301–17.

[dataset]23 LIHVOICE.Predi_COVID_Fatigue_Vocal_Biomarker/Android_audioset.csv at main · LIHVOICE/Predi_COVID_Fatigue_Vocal_Biomarker. GitHub. https://github.com/LIHVOICE/Predi_COVID_Fatigue_Vocal_Biomarker (accessed Jan 31, 2022).

[dataset] 24 Elbéji A, Zhang L, Higa E, *et al.* Audio recordings of COVID-19 positive individuals from the prospective Predi-COVID cohort study with their fatigue status. 2022; published online Feb 1. DOI:10.5281/zenodo.5937844.

25 Barman R, Deshpande S, Agarwal S, Inamdar U, Devare M. Transfer Learning for Small Dataset. 2019; published online March 26. http://dx.doi.org/ (accessed Nov 18, 2021).

26 Weiss K, Khoshgoftaar TM, Wang D. A survey of transfer learning. *Journal of Big Data* 2016; **3**: 1–40.

27 Simonyan K, Zisserman A. Very Deep Convolutional Networks for Large-Scale Image Recognition. 2014; published online Sept 4. http://arxiv.org/abs/1409.1556

For peer review only

For peer review only - http://bmjopen.bmj.com/site/about/guidelines.xhtml

(accessed Aug 6, 2021).

28 Hasan BMS, Abdulazeez AM. A Review of Principal Component Analysis Algorithm for Dimensionality Reduction. *Journal of Soft Computing and Data Mining* 2021; **2**: 20–30.

29 König A, Satt A, Sorin A, *et al.* Automatic speech analysis for the assessment of patients with predementia and Alzheimer's disease. *Alzheimer's & dementia (Amsterdam, Netherlands)* 2015; **1**. DOI:10.1016/j.dadm.2014.11.012.

30 Zhang L, Duvvuri R, Chandra KKL, Nguyen T, Ghomi RH. Automated voice biomarkers for depression symptoms using an online cross-sectional data collection initiative. *Depress Anxiety* 2020; **37**. DOI:10.1002/da.23020.

31 Noninvasive Vocal Biomarker is Associated With Severe Acute Respiratory Syndrome Coronavirus 2 Infection. *Mayo Clinic Proceedings: Innovations, Quality & Outcomes* 2021; **5**: 654–62.

32 COVID-19 Sounds App. http://www.covid-19-sounds.org/ (accessed Nov 18, 2021).

33 Goërtz YMJ, Van Herck M, Delbressine JM, *et al.* Persistent symptoms 3 months after a SARS-CoV-2 infection: the post-COVID-19 syndrome? *ERJ Open Res* 2020; **6**. DOI:10.1183/23120541.00542-2020.

34 Townsend L, Dyer AH, Jones K, *et al.* Persistent fatigue following SARS-CoV-2 infection is common and independent of severity of initial infection. *PLoS One* 2020; **15**: e0240784.

35 Arnold P, Njemini R, Vantieghem S, *et al.* Peripheral muscle fatigue in hospitalised geriatric patients is associated with circulating markers of inflammation. Experimental Gerontology. 2017; **95**: 128–35.

36 Fogarty H, Townsend L, Morrin H, *et al.* Persistent endotheliopathy in the pathogenesis of long COVID syndrome. *J Thromb Haemost* 2021; **19**. DOI:10.1111/jth.15490.

37 Pan S-Y, Ding M, Huang J, Cai Y, Huang Y-Z. Airway resistance variation

For peer review only

For peer review only - http://bmjopen.bmj.com/site/about/guidelines.xhtml

correlates with prognosis of critically ill COVID-19 patients: A computational fluid dynamics study. *Comput Methods Programs Biomed* 2021; **208**: 106257.

38 Murry T. The voice and its disorders, 4th edition. By Margaret C. L. Greene, 446 pp, illus, J. B. Lippincott Co., Philadelphia, PA, 1980. $47.50. 1981. DOI:10.1002/HED.2890030517.

39 Zörner S, Kaltenbacher M, Döllinger M. Investigation of prescribed movement in fluid–structure interaction simulation for the human phonation process. Computers & Fluids. 2013; **86**: 133–40.

40 Yin J, Zhang Z. Laryngeal muscular control of vocal fold posturing: Numerical modeling and experimental validation. *J Acoust Soc Am* 2016; **140**: EL280.

41 Dassie-Leite AP, Gueths TP, Ribeiro VV, Pereira EC, Martins P do N, Daniel CR. Vocal Signs and Symptoms Related to COVID-19 and Risk Factors for their Persistence. *J Voice* 2021; published online Aug 11. DOI:10.1016/j.jvoice.2021.07.013.

Legends

Table 1: Study population characteristics
Table 2: Results of the prediction models
Figure 1. General Pipeline
Figure 2. VGG19 Feature Extraction
Figure 3. Derivation of the digital fatigue vocal biomarker for Android and iOS users.
SOM 1. Text to read
SOM 2. VGG19 extracted features from participants' audio recordings

For peer review only

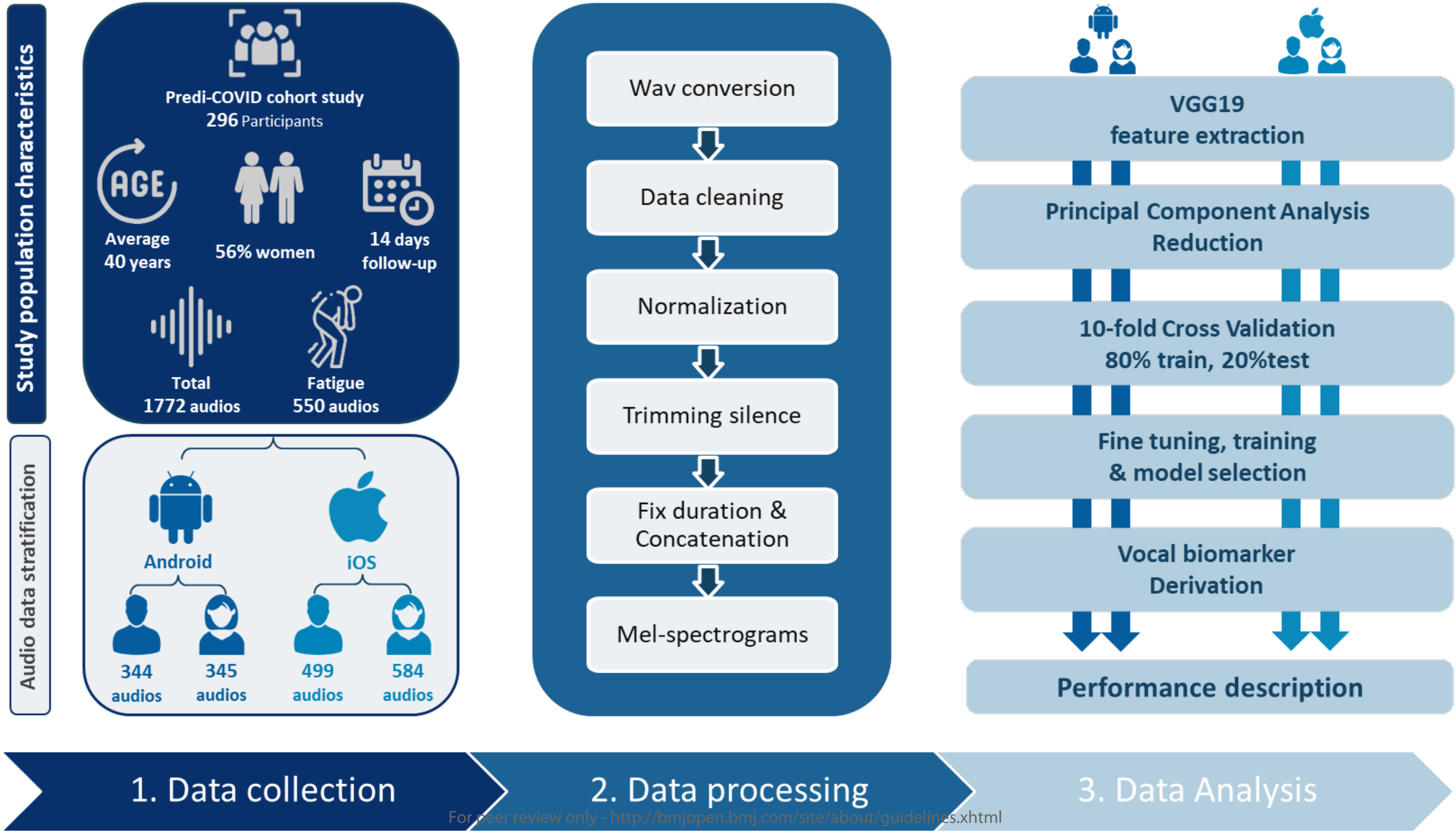
Study population characteristics
Predi-COVID cohort study
296 Participants
Average
40 years
56% women
14 days
follow-up
Total
1772 audios
Fatigue
550 audios
Audio data stratification
Android
iOS
344
audios
345
audios
499
audios
584
audios
Wav conversion
Data cleaning
Normalization
Trimming silence
Fix duration &
Concatenation
Mel-spectrograms
VGG19
feature extraction
Principal Component Analysis
Reduction
10-fold Cross Validation
80% train, 20%test
Fine tuning, training
& model selection
Vocal biomarker
Derivation
Performance description
1. Data collection
2. Data processing
3. Data Analysis

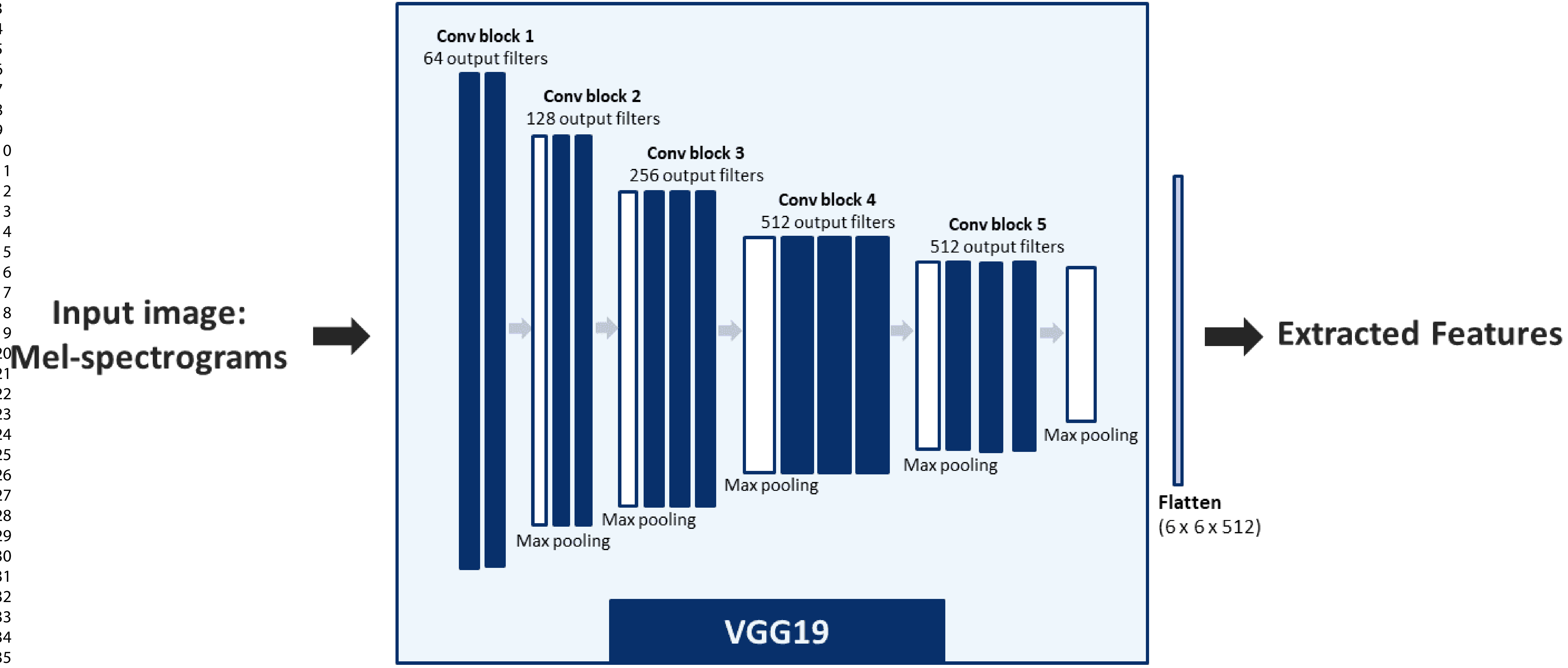
Input image: Mel-spectrograms
Conv block 1
64 output filters
Conv block 2
128 output filters
Conv block 3
256 output filters
Conv block 4
512 output filters
Conv block 5
512 output filters
Max pooling
Max pooling
Max pooling
Max pooling
Max pooling
VGG19
Flatten
(6 x 6 x 512)
Extracted Features

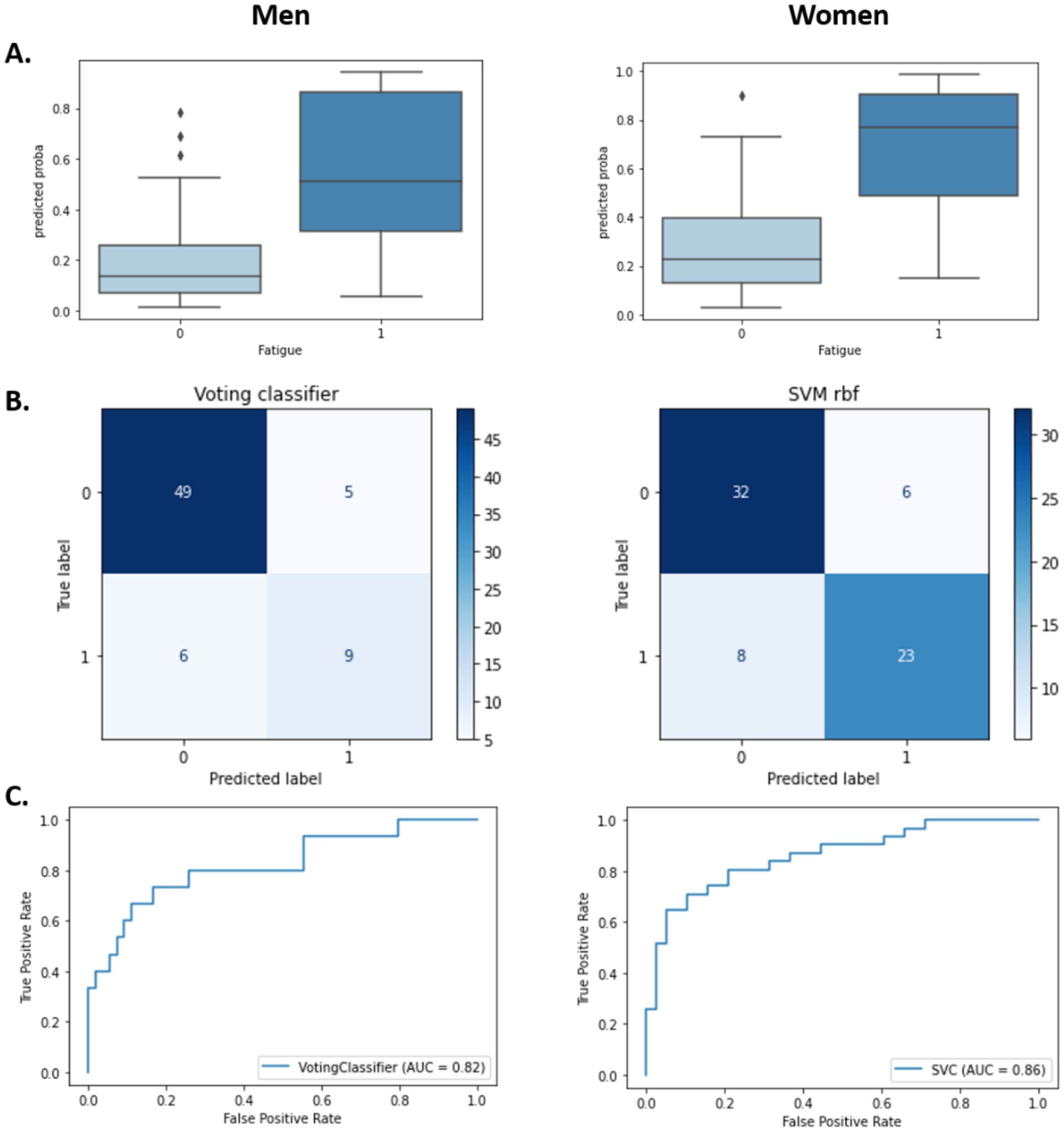

Figure 3a. Derivation of the digital fatigue vocal biomarker for Android users

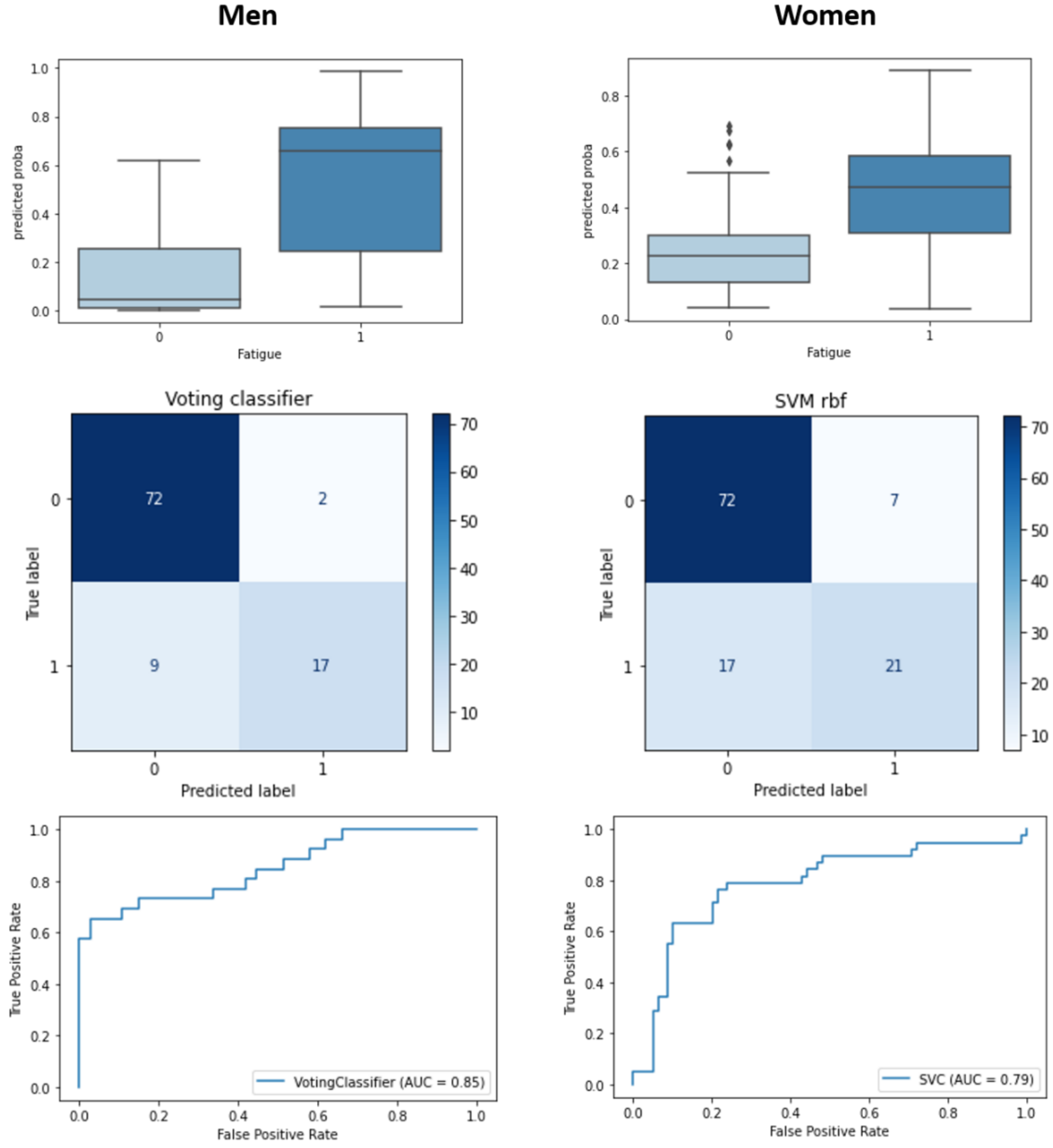

Figure 3b. Derivation of the digital fatigue vocal biomarker for iOS users

Supplementary Online Material 1. Standardized, prespecified text to be read by study participants to collect voice recordings.

Universal Declaration of Human Rights, United Nations.

English

Everyone has the right to a standard of living adequate for the health and well-being of himself and of his family, including food, clothing, housing and medical care and necessary social services, and the right to security in the event of unemployment, sickness, disability, widowhood, old age or other lack of livelihood in circumstances beyond his control.

French

Toute personne a droit à un niveau de vie suffisant pour assurer sa santé, son bien-être et ceux de sa famille, notamment pour l'alimentation, l'habillement, le logement, les soins médicaux ainsi que pour les services sociaux nécessaires ; elle a droit à la sécurité en cas de chômage, de maladie, d'invalidité, de veuvage, de vieillesse ou dans les autres cas de perte de ses moyens de subsistance par suite de circonstances indépendantes de sa volonté.

German

Jeder hat das Recht auf einen Lebensstandard, der seine und seiner Familie Gesundheit und Wohl gewährleistet, einschließlich Nahrung, Kleidung, Wohnung,

ärztliche Versorgung und notwendige soziale Leistungen gewährleistet sowie das Recht auf Sicherheit im Falle von Arbeitslosigkeit, Krankheit, Invalidität oder Verwitwung, im Alter sowie bei anderweitigem Verlust seiner Unterhaltsmittel durch unverschuldete Umstände.

Portuguese

Toda a pessoa tem direito a um nível de vida suficiente para lhe assegurar e à sua família a saúde e o bem-estar, principalmente quanto à alimentação, ao vestuário, ao alojamento, à assistência médica e ainda quanto aos serviços sociais necessários, e tem direito à segurança no desemprego, na doença, na invalidez, na viuvez, na velhice ou noutros casos de perda de meios de subsistência por circunstâncias independentes da sua vontade.

Supplementary Online Material 2. VGG19 extracted features from participants' audio recordings

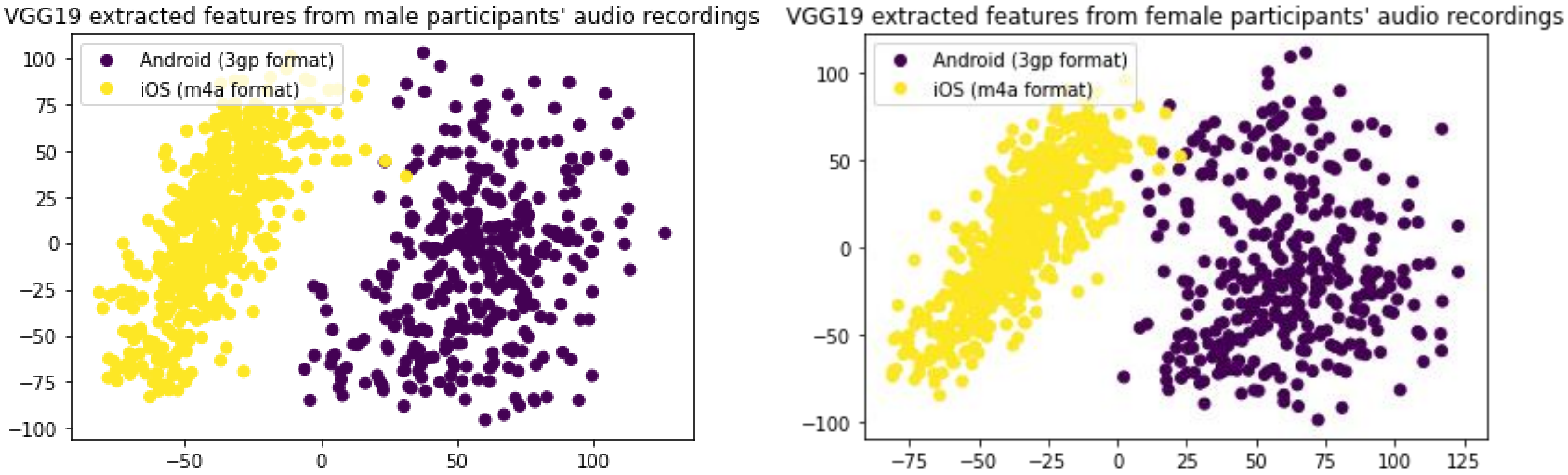

The scatter plot of the 250 relevant components given by PCA reduction revealed two distinct clusters. These two groups appeared to characterize the audio formats, m4a (iOS users) and 3gp (Android users).

It was consequently hypothesized that our data was heterogeneous and that it would be preferable to fit the models with each audio format independently.

TRIPOD Checklist: Prediction Model Development and Validation

Section/Topic	Item		Checklist Item	Page
**Title and abstract**				
Title	1	D;V	Identify the study as developing and/or validating a multivariable prediction model, the target population, and the outcome to be predicted.	1
Abstract	2	D;V	Provide a summary of objectives, study design, setting, participants, sample size, predictors, outcome, statistical analysis, results, and conclusions.	2
**Introduction**				
Background and objectives	3a	D;V	Explain the medical context (including whether diagnostic or prognostic) and rationale for developing or validating the multivariable prediction model, including references to existing models.	4
	3b	D;V	Specify the objectives, including whether the study describes the development or validation of the model or both.	5
**Methods**				
Source of data	4a	D;V	Describe the study design or source of data (e.g., randomized trial, cohort, or registry data), separately for the development and validation data sets, if applicable.	5
	4b	D;V	Specify the key study dates, including start of accrual; end of accrual; and, if applicable, end of follow-up.	
Participants	5a	D;V	Specify key elements of the study setting (e.g., primary care, secondary care, general population) including number and location of centres.	6
	5b	D;V	Describe eligibility criteria for participants.	
	5c	D;V	Give details of treatments received, if relevant.	
Outcome	6a	D;V	Clearly define the outcome that is predicted by the prediction model, including how and when assessed.	9
	6b	D;V	Report any actions to blind assessment of the outcome to be predicted.	
Predictors	7a	D;V	Clearly define all predictors used in developing or validating the multivariable prediction model, including how and when they were measured.	8
	7b	D;V	Report any actions to blind assessment of predictors for the outcome and other predictors.	
Sample size	8	D;V	Explain how the study size was arrived at.	7
Missing data	9	D;V	Describe how missing data were handled (e.g., complete-case analysis, single imputation, multiple imputation) with details of any imputation method.	
Statistical analysis methods	10a	D	Describe how predictors were handled in the analyses.	8
	10b	D	Specify type of model, all model-building procedures (including any predictor selection), and method for internal validation.	
	10c	V	For validation, describe how the predictions were calculated.	
	10d	D;V	Specify all measures used to assess model performance and, if relevant, to compare multiple models.	
	10e	V	Describe any model updating (e.g., recalibration) arising from the validation, if done.	
Risk groups	11	D;V	Provide details on how risk groups were created, if done.	
Development vs. validation	12	V	For validation, identify any differences from the development data in setting, eligibility criteria, outcome, and predictors.	9
**Results**				
Participants	13a	D;V	Describe the flow of participants through the study, including the number of participants with and without the outcome and, if applicable, a summary of the follow-up time. A diagram may be helpful.	9
	13b	D;V	Describe the characteristics of the participants (basic demographics, clinical features, available predictors), including the number of participants with missing data for predictors and outcome.	
	13c	V	For validation, show a comparison with the development data of the distribution of important variables (demographics, predictors and outcome).	
Model development	14a	D	Specify the number of participants and outcome events in each analysis.	9
	14b	D	If done, report the unadjusted association between each candidate predictor and outcome.	
Model specification	15a	D	Present the full prediction model to allow predictions for individuals (i.e., all regression coefficients, and model intercept or baseline survival at a given time point).	10
	15b	D	Explain how to the use the prediction model.	
Model performance	16	D;V	Report performance measures for the prediction model.	10
Model-updating	17	V	If done, report the results from any model updating (i.e., model specification, model performance).	
**Discussion**				
Limitations	18	D;V	Discuss any limitations of the study (such as nonrepresentative sample, few events per predictor, missing data).	11
Interpretation	19a	V	For validation, discuss the results with reference to performance in the development data, and any other validation data.	10
	19b	D;V	Give an overall interpretation of the results, considering objectives, limitations, results from similar studies, and other relevant evidence.	
Implications	20	D;V	Discuss the potential clinical use of the model and implications for future research.	12
**Other information**				
Supplementary information	21	D;V	Provide information about the availability of supplementary resources, such as study protocol, Web calculator, and data sets.	5,6,10
Funding	22	D;V	Give the source of funding and the role of the funders for the present study.	3

*Items relevant only to the development of a prediction model are denoted by D, items relating solely to a validation of a prediction model are denoted by V, and items relating to both are denoted D;V. We recommend using the TRIPOD Checklist in conjunction with the TRIPOD Explanation and Elaboration document.

For peer review only
